# Differences across cyclophilin A orthologs contribute to the host range restriction of hepatitis C virus

**DOI:** 10.7554/eLife.44436

**Published:** 2019-05-10

**Authors:** Jenna M Gaska, Metodi Balev, Qiang Ding, Brigitte Heller, Alexander Ploss

**Affiliations:** 1 Department of Molecular BiologyPrinceton UniversityPrincetonUnited States; Ulm University Medical CenterGermany; Utrecht UniversityNetherlands

**Keywords:** Hepatitis C virus, species tropism, animal model, Human, Mouse, Virus

## Abstract

The restricted host tropism of hepatitis C virus (HCV) remains incompletely understood, especially post-entry, and has hindered developing an immunocompetent, small animal model. HCV replication in non-permissive species may be limited by incompatibilities between the viral replication machinery and orthologs of essential host factors, like cyclophilin A (CypA). We thus compared the ability of CypA from mouse, tree shrew, and seven non-human primate species to support HCV replication, finding that murine CypA only partially rescued viral replication in Huh7.5-shRNA CypA cells. We determined the specific amino acid differences responsible and generated mutants able to fully rescue replication. We expressed these mutants in engineered murine hepatoma cells and although we observed increases in HCV replication following infection, they remained far lower than those in highly permissive human hepatoma cells, and minimal infectious particle release was observed. Together, these data suggest additional co-factors remain unidentified. Future work to determine such factors will be critical for developing an immunocompetent mouse model supporting HCV replication.

## eLife digest

Hepatitis C is a life-long disease that begins when a virus infects the cells of the liver. Although the infection is curable, it is expensive to treat, and there is not yet a vaccine to prevent the disease. This is largely because the virus that causes hepatitis C, also known as HCV, naturally only infects humans and chimpanzees, which has made it difficult to generate an effective animal model for developing a vaccine.

Mice are frequently used as a model for studying disease and can be genetically altered to allow HCV to enter their liver cells. However, once HCV enters mouse cells, it struggles to replicate. As a result, an infection does not develop, and the immune system’s response to the virus cannot be fully explored. Replication of HCV in humans relies on a protein called cyclophilin A, or CypA for short. Now, Gaska et al. have set out to improve current animal models for HCV by investigating whether HCV can also use CypA from other species, including mice, to replicate.

Gaska et al. showed that the mouse form of CypA could help HCV replicate in human liver cells with lower than normal levels of CypA, but only very poorly. Editing the mouse gene for CypA to be more like the human version resulted in higher HCV replication. Putting variants of CypA into the liver cells of mice, which do not normally replicate HCV at high levels, led to an increase in HCV replication. However, replication of HCV in mice was still far lower than in human liver cells, suggesting that the mouse model system could be improved by learning more about which proteins interact with CypA.

Injection drug use – one of the main ways hepatitis C spreads – is becoming increasingly common because of the growing opioid epidemic in many countries. A clinically relevant animal model that supports hepatitis C virus infection would be an important milestone towards a vaccine that could prevent the continued spread of this disease.

## Introduction

Every year, an estimated 3–4 million individuals become newly infected with hepatitis C virus (HCV) (Westbrook and Dusheiko, 2014), with 60–80% going on to join the population of approximately 71.1 million who are chronically infected (Blach et al., 2017). A hepatotropic virus, HCV has only been shown to robustly infect in vivo human and chimpanzee hepatocytes. This limited host tropism has proven problematic in developing an animal model that is not only ethically and financially sound but also immunocompetent. Such a model would allow the study of the underlying immunopathogenesis of HCV infection as well as the development and testing of vaccine candidates. Most efforts to generate such an in vivo model have focused on mice, which are amenable to genetic manipulation and have many well-established research tools built around their use.

Overcoming the natural imperviousness of murine hepatocytes to HCV has required adjustments at multiple stages of the virus life cycle. Barriers at the level of entry (McCaffrey et al., 2002; Park et al., 2009) in murine cells could not be overcome until the identification of the four canonical HCV human entry factors – claudin-1 (CLDN1; Evans et al., 2007), occludin (OCLN; Liu et al., 2009a; Ploss et al., 2009), CD81 (Pileri et al., 1998) and scavenger receptor class B member 1 (SCARB1; Scarselli et al., 2002). Of these, human CD81 and human OCLN were the minimal factors needed for viral entry (Ploss et al., 2009). Alternatively, infecting with HCV adapted to utilize murine CD81 could also successfully overcome this initial obstacle (Bitzegeio et al., 2010). Once inside murine cells, HCV faces another block at the level of replication, which initial studies circumvented through the use of selectable subgenomic HCV replicons (McCaffrey et al., 2002; Park et al., 2009; Uprichard et al., 2006). Murine cells did appear to support assembly and release of infectious particles, albeit at low levels, once NS2 and the structural HCV proteins were provided in trans and the murine or human ortholog of the apolipoprotein ApoE was overexpressed (Long et al., 2011). Further efforts to improve HCV replication in a murine context have relied on disruption of innate immune responses (Aly et al., 2011; Anggakusuma et al., 2015; Chang et al., 2006; Frentzen et al., 2014; Lin et al., 2010; Nandakumar et al., 2013), with robust replication and completion of the HCV life cycle proving difficult following infection unless a selectable genome is used (Vogt et al., 2013).

These poor levels of replication could be due to the lower compatibility of murine orthologs of vital replication host factors with the viral replication machinery or the absence of proteins that normally facilitate these interactions in human cells. Although numerous intracellular host factors have been implicated in HCV replication, extensive experimental evidence has only been provided for three host factors: cyclophilin A (CypA) (Kaul et al., 2009; Yang et al., 2008a), phosphatidylinositol four kinase IIIα (PI4KA) (Berger et al., 2009; Borawski et al., 2009; Reiss et al., 2011; Tai et al., 2009; Trotard et al., 2009) and microRNA-122 (miR-122) (Jopling et al., 2005; Lanford et al., 2010; Machlin et al., 2011). miR-122 is highly conserved between humans and other species, including mice, making its expression alone unlikely to explain the weak replication of HCV in murine cells. In an effort to systemically dissect the impact of such replication co-factors during infection, we focused in this study on CypA and how it may contribute to the restricted host range of HCV.

CypA is a cytosolic 18 kDa peptidyl-prolyl *cis-trans* isomerase (PPIase) and a part of the biologically ubiquitous cyclophilin enzyme family (Fischer et al., 1989), the members of which were first characterized in mammals by their common ability to bind the immunosuppressive drug cyclosporin A (CsA) and their shared cyclophilin-like domain (CLD) which catalyzes the *cis-trans* isomerization of proline residues (reviewed in Marks, 1996). CypA overexpression has been implicated in a wide variety of human diseases, ranging from cancer to atherosclerosis (reviewed in Nigro et al., 2013), and it has a demonstrated role in the life cycles of multiple viruses besides HCV (de Wilde et al., 2018; Frausto et al., 2013; Li et al., 2016; Phillips et al., 2015; Tian et al., 2010; von Hahn and Ciesek, 2015; Watashi and Shimotohno, 2007; Zhou et al., 2012). Early work showed that CsA had an inhibitory effect on HCV in chronically infected chimpanzees, but it was not until subsequent in vitro CypA knockdown experiments and dose-response assays with CsA derivatives that CypA was specifically recognized as critical to HCV replication (Chatterji et al., 2009; Ciesek et al., 2009; Coelmont et al., 2009; Kaul et al., 2009; Liu et al., 2009b; Yang et al., 2008b).

These studies showed that CypA’s relevance to HCV replication was intimately linked to its PPIase activity, as the introduction of point mutations in the PPIase active site led to impaired viral replication (Chatterji et al., 2009; Kaul et al., 2009; Liu et al., 2009b). Individuals exhibiting an HCV non-permissive phenotype were shown to express a rare homozygosity at any of three SNP sites in the coding region of CypA – but not in the enzymatic active site – that subsequent in vitro work showed resulted in markedly decreased levels of intracellular CypA (von Hahn et al., 2012). Despite its known importance, the exact mechanism by which CypA facilitates HCV replication remains poorly characterized. Interactions between CypA and several HCV proteins have been demonstrated, including the RNA-dependent RNA polymerase (RdRP) NS5B (Chatterji et al., 2009; Fernandes et al., 2007; Robida et al., 2007; Yang et al., 2008b), NS5A (Anderson et al., 2011; Coelmont et al., 2010; Foster et al., 2011; Grisé et al., 2012; Hanoulle et al., 2009; Nag et al., 2012; Verdegem et al., 2011) and NS2 (Ciesek et al., 2009; Kaul et al., 2009), but how CypA’s binding, PPIase activity and the viral polyprotein are precisely intertwined remains to be understood. The specific impact that cross-species differences in CypA might have on HCV replication and the restricted host tropism of this virus remains an open question and one the present study sought to address. Here, we examined the ability of CypA from diverse species, some of which could serve as feasible small animal models for HCV, to facilitate HCV replication. We found that murine CypA, relative to human CypA, is less proficient at facilitating HCV replication due to differences at the amino acid level and that overexpression of human CypA can increase replication in an engineered murine hepatoma line.

## Results

### Ability of diverse CypA orthologs to facilitate HCV replication

Knowing the critical role of human CypA in facilitating HCV replication, we first examined the conservation of CypA at the amino acid level across diverse species, focusing on those with promise to serve as biomedical research models and/or closely related to humans. As observed in mice, previous in vivo studies have suggested that several non-human primate (NHP) species – including cynomolgus, Japanese, and rhesus macaque; African green monkey; and Chacma and doguera baboons – appear resistant to HCV infection (Abe et al., 1993; Bukh et al., 2001). In contrast, more recent work in vitro demonstrated that primary hepatocytes from rhesus macaques (PRMH) (Scull et al., 2015) as well as hepatocyte-like cells derived from pigtailed macaque induced pluripotent stem cells (iPSCs) could support the HCV life cycle (Sourisseau et al., 2013). Importantly, pharmacological-mediated suppression of innate immune responses via Jak inhibition enhanced viral replication in PRMH (Scull et al., 2015). Additionally, albeit with limited evidence, tree shrews have also been demonstrated as a potential platform for studying HCV infection (Amako et al., 2010; Tong et al., 2011; Xie et al., 1998; Xu et al., 2007).

**Figure 1.**
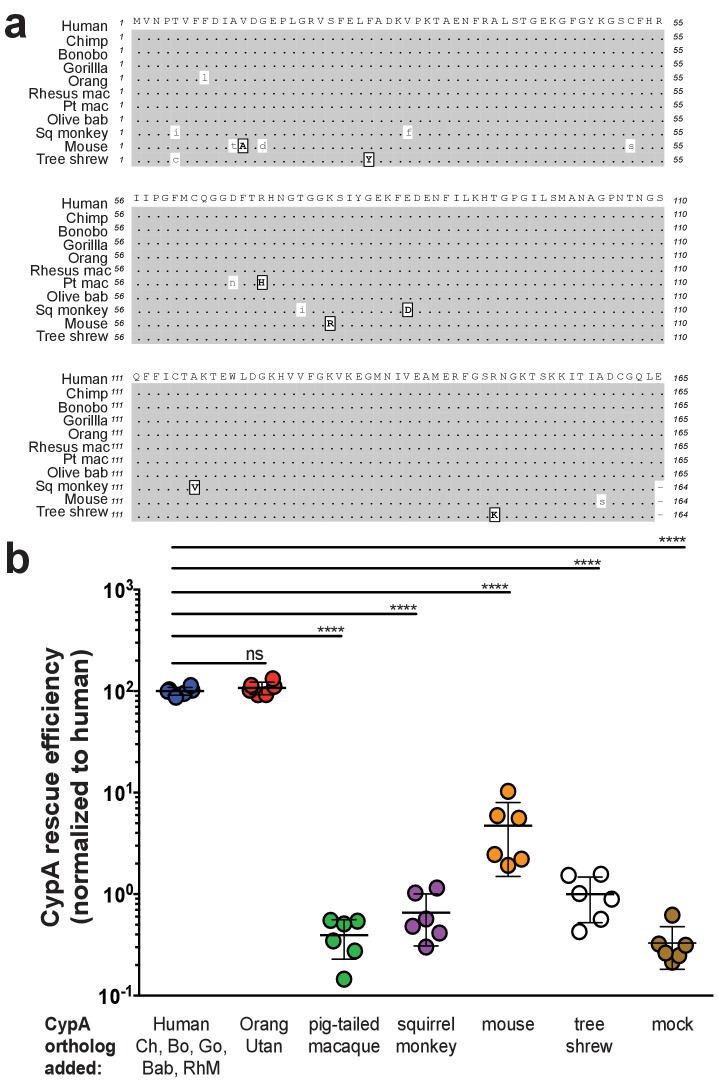
Murine CypA has a diminished ability to facilitate HCV replication. (**A**) An amino acid sequence alignment of CypA from diverse species. Similar amino acids are indicated in boxed, bold capital letters while differences are lowercase. Species are arranged from top to bottom in increasing evolutionary distance from human. For pigtailed macaque, all experiments utilized a TRIM5-CypA fusion – only the residues of the CypA portion of the fusion are depicted here. (**B**) Huh7.5 cells expressing an shRNA against endogenous human CypA (Huh7.5-shRNA CypA) were transduced to express different CypA orthologs and then infected with a HCV reporter genome expressing secreted *Gaussia* luciferase (Jc1-Gluc, MOI = 0.1). At five dpi, the luciferase activity of the supernatants was assessed as a proxy for viral replication. CypA rescue efficiency is shown normalized to Huh7.5-shRNA CypA transduced with human CypA, which is 100% identical at the amino acid level to chimpanzee, bonobo, gorilla, olive baboon and rhesus macaque CypA. Results shown are from two representative experiments, each with triplicate samples. Lines and error bars represent the mean ± SD. Ordinary two-way ANOVA test performed followed by Dunnett’s multiple comparisons test with all means compared to that of the +human CypA line. Chimp/Ch, chimpanzee; Bo, bonobo; Go, gorilla; Orang, orangutan; Rhesus mac/RhM, rhesus macaque; Pt mac, pigtailed macaque; Olive bab/Bab, olive baboon; Sq monkey, squirrel monkey. ****, p<0.0001; ns, not significant.

Thus, in the present study we compared the amino acid similarity of CypA from great apes (human, chimpanzee, bonobo, gorilla, orangutan), Old World monkeys (rhesus macaque, pigtailed macaque, olive baboon), a New World monkey (squirrel monkey), tree shrew, and mouse (Figure 1a). Human, chimpanzee, bonobo, gorilla, olive baboon, and rhesus macaque CypA are 100% identical at the amino acid level and for subsequent experiments the human CypA (hCypA) CDS was used as the representative sequence for these six species. Multiple studies have shown that pigtailed macaques are predominantly homozygous for an insertion of the CypA exon at the *TRIM5* locus, resulting in a chimeric TRIM5-CypA transcript (Brennan et al., 2008; Liao et al., 2007; Newman et al., 2008), which we used for our experiments.

**Figure 1—figure supplement 1.**
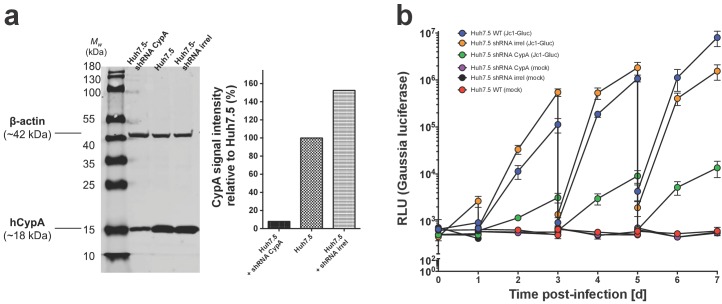
Assessment of Huh7.5-shRNA CypA cells. (**A**) Western blot demonstrating knockdown of human CypA in Huh7.5-shRNA CypA cells relative to the parental Huh7.5 cells and those expressing an shRNA against an irrelevant target (Huh7.5-shRNA irrel). Quantification of signal intensity was done using LI-COR Image Studio Software (version 4.0) with background detection set to ‘Median.’ CypA signal intensity was normalized based off that of β-actin and is expressed here relative to Huh7.5 cells (set at 100%). (**B**) HCV replication is specifically diminished in Huh7.5-shRNA CypA cells and not Huh7.5-shRNA irrel cells. Infection performed with Jc1-Gluc (MOI = 0.1). On even-numbered days, cells were washed with PBS and the media changed to assess de novo replication. Lines and error bars represent the mean ± SD.

**Figure 1—figure supplement 2.**
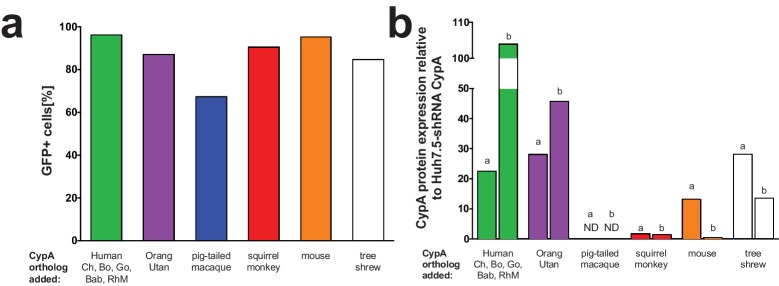
Transduction efficiency of CypA orthologs in Huh7.5-shRNA CypA cells. Bicistronic constructs expressing the CypA orthologs of interest followed by an IRES-regulated eGFP-ubiquitin-neomycin fusion protein were used to transduce Huh7.5-shRNA CypA cells. Transduction efficiency assessed via flow cytometry and representative data are summarized here from one set of transductions (**A**). Cell lysates were collected from Huh7.5-shRNA CypA cells transduced with these various constructs and expression determined by western blot (please see Supplementary file 1). Quantification of CypA expression was determined by taking the signal from the CypA band divided by the signal from the β-actin band and then this value divided by that from the untransduced Huh7.5-shRNA CypA cells to give the relative expression (**B**). Signal intensity was determined using LI-COR Image Studio Software (version 4.0) with background detection set to ‘Lane.’ The ‘a’ and ‘b’ above each bar delineate the different pairs of antibodies used to assess CypA expression. For ‘a,’ the signal was quantified from rabbit anti-CypA and mouse anti-β-actin; for ‘b,’ the signal was quantified from mouse anti CypA and rabbit anti- β-actin. ND, non-detected; Mn, pigtailed macaque.

Having identified these differences between CypA orthologs, we then compared their respective abilities to support HCV replication in a Huh7.5 cell line stably expressing an shRNA against endogenous human CypA (Huh7.5-shRNA CypA) (von Hahn et al., 2012) (Figure 1—figure supplement 1). Cells were transduced with a bicistronic lentivirus to express the CypA ortholog and a GFP-ubiquitin-neomycin resistance (GUN) fusion protein. The bicistronically expressed GFP provides a straightforward means to monitor protein expression indirectly. We deliberately chose not to add an epitope tag onto the different CypA variants to avoid impacting function. The percentage of GFP+ cells as determined by flow cytometry indicated >60% transduction efficiency (Figure 1—figure supplement 2a). We also assessed protein expression by western blot using two different antibodies with different CypA antigen specificities – commercial antibodies with listed reactivity for human and mouse CypA were available but not for any of the other species under examination (Figure 1—figure supplement 2b, Supplementary file 1). All the orthologs were readily detected at the expected size of ~17 kDa except for squirrel monkey CypA, the signal for which was <2 times that of the background levels in the nontransduced Huh7.5-shRNA CypA cells, and pigtailed macaque CypA. In the latter case, the TRIM5-CypA fusion protein was expected at ~51 kDa but no signal was observed with either antibody. The transduced cells were subsequently infected with the HCV reporter virus Jc1-Gluc (Marukian et al., 2008) at an MOI of 0.1. Levels of *Gaussia* luciferase in the culture supernatants were thus used as a proxy for assessing HCV replication to compare the rescue efficiencies of the CypA orthologs. As expected, expression of human CypA in Huh7.5-shRNA CypA cells increased HCV replication by more than two logs relative to the non-rescued cells at five days post-infection (dpi) (Figure 1b). Of the orthologs tested, only orangutan CypA, which differs from human CypA by a single amino acid, was capable of rescuing HCV replication at levels similar to human. Compared to human CypA (normalized to 100%), mouse CypA could still facilitate HCV replication but at levels ~3–4% of those observed for human, that is ca. 30-fold decrease. Tupaia CypA, which was well expressed by western blot, did not significantly increase HCV replication above the levels observed in the parental Huh7.5-shRNA CypA cells. It remains possible that the lack of HCV replication in cells transduced with squirrel monkey and pigtailed macaque CypA is due to these proteins not being properly expressed. However, as the transduction efficiency of our constructs expressing pigtailed macaque and squirrel monkey CypA was robust and we were able to detect all other CypA variants utilized in this study by western blot, it is more likely that the antibody reactivity for these two specific orthologs is weaker.

### Identifying the amino acid basis for the decreased ability of mouse CypA to facilitate HCV replication

**Figure 2.**
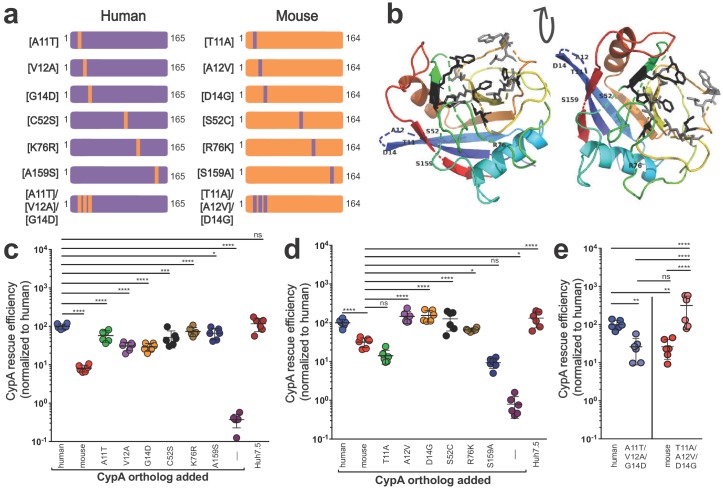
Characterizing the amino acid basis for the differing efficiencies of murine and human CypA in HCV replication. (**A**) Schematic depicting the humanized murine CypA and murinized human CypA constructs tested. (**B**) Modeled structure of human CypA (PDB 1CWA) with the six residues differing between murine and human CypA altered to those of murine CypA and shown labeled. The residues that directly interact with cyclosporine A (CsA) (Arg55, Phe60, Met61, Gln63, Gly72, Ala101, Asn102, Ala103, Gln111, Phe113, Trp121, Leu122 and His126) (Ke et al., 1994) are depicted in gray as stick models. The six residues that comprise the active site (His54, Arg55, Phe60, Gln111, Phe113, and His126) (Zydowsky et al., 1992), five of which also interact with CsA, are shown in black as stick models. Huh7.5-shRNA CypA cells were transduced with the singly murinized human (**C**), the singly humanized murine (**D**) or the triply murinized/humanized (**E**) CypA mutants, infected with Jc1-Gluc at MOI = 0.1 and supernatants assessed for *Gaussia* luciferase activity as a proxy for HCV replication at five dpi. The rescue efficiency of each mutant was normalized to Huh7.5-shRNA CypA cells transduced with human CypA. Results shown are from two representative experiments, each with triplicate samples. Lines and error bars represent the mean ± SD. Ordinary two-way ANOVA test performed followed by Sidak’s multiple comparison test, with all means compared to that of the +human CypA line (**C**) or the +mouse CypA line (**D**). For (**E**), Tukey's multiple comparison test was used to compare all the means to one another. *, p<0.05; **, p<0.01; ***, p<0.001; ****, p<0.0001; ns, not significant.

As known blocks in the viral life cycle at the level of entry have been well characterized in murine hepatocytes, we aimed to further understand how murine CypA might affect viral replication. Thus, still in a human context, we sought to determine how the six amino acid differences between murine and human CypA contributed to the decreased ability of murine CypA to facilitate HCV replication in Huh7.5-shRNA CypA cells. ‘Murinized’ human CypA and ‘humanized’ murine CypA mutants were generated whereby each of the six differing amino acids were changed one at a time to their murine or their human counterpart, respectively (Figure 2a), and transduction (Figure 2—figure supplement 1) as well as CypA expression confirmed (Figure 2—figure supplement 2, Supplementary file 1). None of these differences fell in the CsA-binding site of CypA (Figure 2b). Human CypA was more sensitive to changes in the amino acid sequence, with significant decreases in rescue efficiency for all single residue changes tested (Figure 2c). Mouse CypA demonstrated a greater ability to facilitate HCV replication, at least in a human cell context, when either residues 12, 14, 52 or 76 were altered, with levels of replication comparable to those observed in the presence of human CypA (Figure 2d). As three of the residues that differ between mouse and human CypA are clustered together (residues 11, 12 and 14), we also constructed and tested mutants triply ‘humanized’ or ‘murinized’ at these positions. Indeed, we observed a striking reversal of phenotype for both constructs, with ‘humanized’ murine CypA able to rescue HCV replication at levels comparable to human and vice versa for ‘murinized’ human CypA (Figure 2e).

**Figure 2—figure supplement 1.**
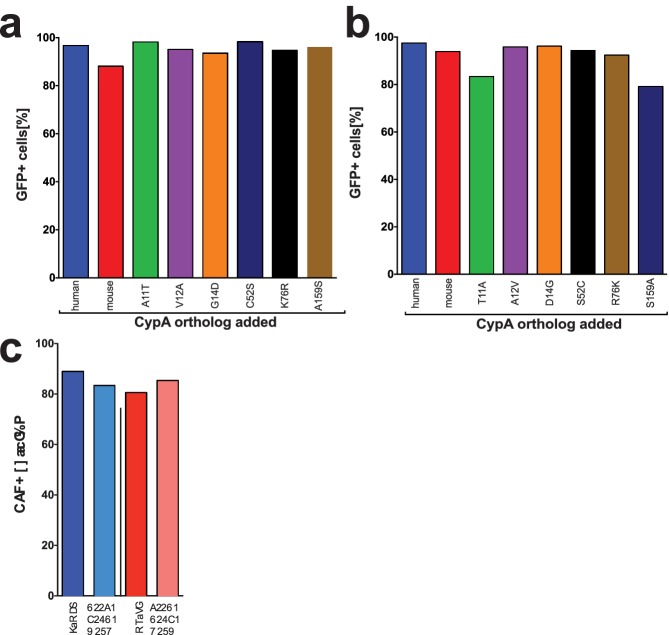
Transduction efficiency of CypA mutants in Huh7.5-shRNA CypA cells. Bicistronic constructs expressing the CypA mutants shown in Figure 2A followed by an IRES-regulated eGFP-ubiquitin-neomycin fusion protein were used to transduce Huh7.5-shRNA CypA cells. Transduction efficiency assessed via flow cytometry and representative data are summarized here from one set of transductions for the murinized human CypA single mutants (**A**), humanized mouse CypA single mutants (**B**) and the triply humanized/triply murinized CypA mutants (**C**).

**Figure 2—figure supplement 2.**
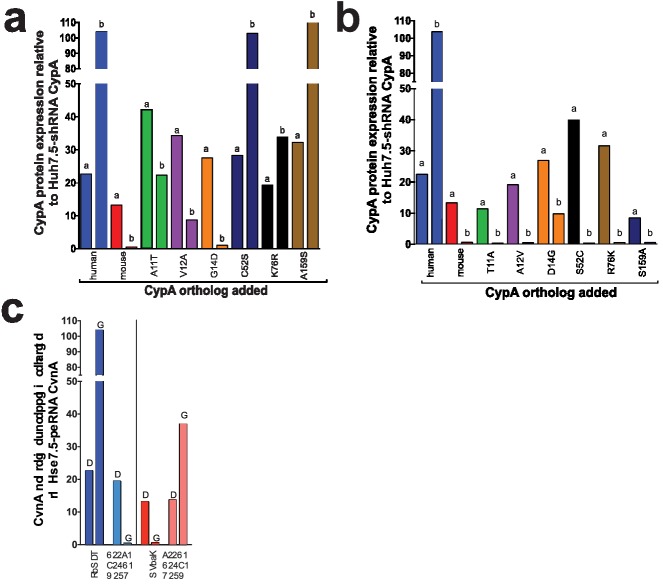
Expression of CypA mutants in Huh7.5-shRNA CypA cells. Cell lysates were collected from the transduced Huh7.5-shRNA CypA cells shown in Figure 2 and Figure 2—figure supplement 1 and expression determined by western blot (please see Supplementary file 1) transductions for the murinized human CypA single mutants (**A**), humanized mouse CypA single mutants (**B**) and the triply humanized/triply murinized CypA mutants (**C**). Quantification of CypA expression was determined by taking the signal from the CypA band divided by the signal from the β-actin band and then this value divided by that from the untransduced Huh7.5-shRNA CypA cells to give the relative expression. Signal intensity was determined using LI-COR Image Studio Software (version 4.0) with background detection set to ‘Lane.’ The ‘a’ and ‘b’ above each bar delineate the different pairs of antibodies used to assess CypA expression. For ‘a,’ the signal was quantified from rabbit anti-CypA and mouse anti-β-actin; for ‘b,’ the signal was quantified from mouse anti CypA and rabbit anti- β-actin.

**Figure 3.**
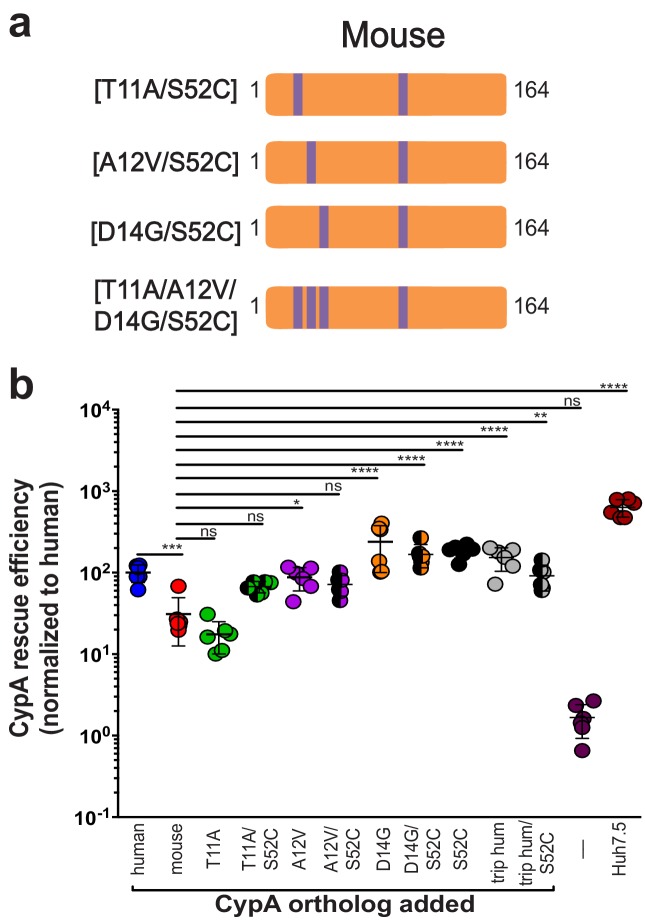
Humanizing residue 52 in the murine CypA mutant T11A/A12V/D14G does not further increase rescue efficiency. (**A**) Schematic of the additional humanized mouse CypA mutants tested. (**B**) Huh7.5-shRNA CypA cells were transduced with the mutants shown in (**A**) and infected with Jc1-Gluc at MOI = 0.1. Supernatants were assessed for *Gaussia* luciferase activity as a proxy for HCV replication at five dpi, and the rescue efficiency of each mutant was normalized to Huh7.5-shRNA CypA transduced with human CypA. Results shown are from two representative experiments, each with triplicate samples. Lines and error bars represent the mean ± SD. Ordinary two-way ANOVA test performed followed by Sidak’s multiple comparison test, with all means compared to that of the +mouse CypA line. *, p<0.05; **, p<0.01; ***, p<0.001; ****, p<0.0001, ns, not significant.

Since humanizing residue 52 in murine CypA also resulted in a strong and significant increase in HCV replication, we combined the S52C mutant with each of the individual mutants T11A, A12V, and D14G as well as with the triply humanized mouse mutant T11A/A12V/D14G to ascertain whether there was an additional increase in HCV replication (Figure 3a, Figure 3—figure supplement 1, Supplementary file 1). Mutating residue 52, even in the triple mutant, did not have a significant synergistic effect for any of the mutants tested (Figure 3b). Compared to mutant T11A, mutant T11A/S52C did demonstrate increased rescue efficiency, but upon performing further statistical tests to those shown in the figure, this was not statistically significant.

**Figure 3—figure supplement 1.**
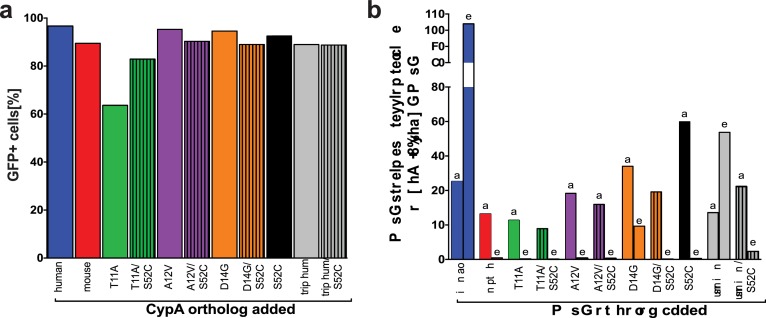
Transduction efficiency of additional mouse-S52C CypA mutants in Huh7.5-shRNA CypA cells. Bicistronic constructs expressing the CypA mutants shown in Figure 3A followed by an IRES-regulated eGFP-ubiquitin-neomycin fusion protein were used to transduce Huh7.5-shRNA CypA cells. Transduction efficiency assessed via flow cytometry and representative data are summarized here from one set of transductions (**A**). Cell lysates were collected from Huh7.5-shRNA CypA cells transduced with these various constructs and expression determined by western blot (please see Supplementary file 1). Quantification of CypA expression was determined by taking the signal from the CypA band divided by the signal from the β-actin band and then this value divided by that from the untransduced Huh7.5-shRNA CypA cells to give the relative expression (**B**). Signal intensity was determined using LI-COR Image Studio Software (version 4.0) with background detection set to ‘Lane.’ The ‘a’ and ‘b’ above each bar delineate the different pairs of antibodies used to assess CypA expression. For ‘a,’ the signal was quantified from rabbit anti-CypA and mouse anti-β-actin; for ‘b,’ the signal was quantified from mouse anti CypA and rabbit anti- β-actin.

### Murine CypA does not exhibit a dominant negative effect on HCV replication

**Figure 4.**
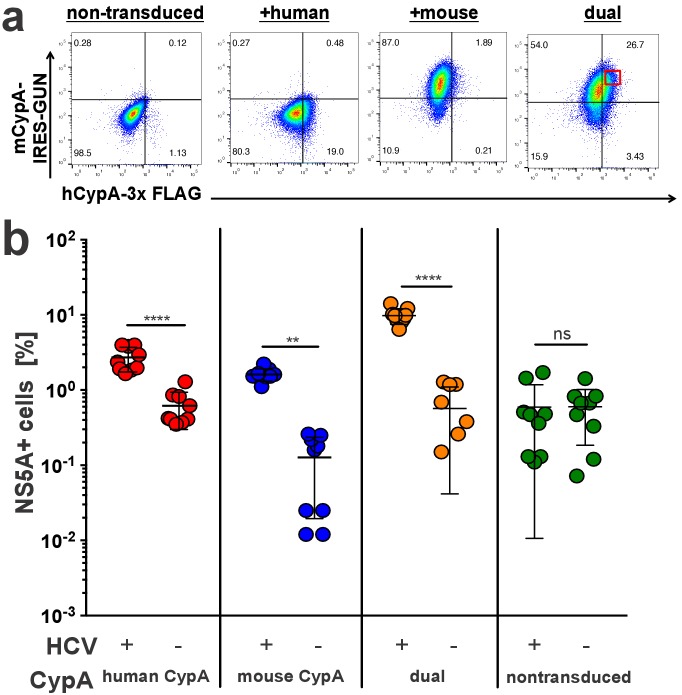
Mouse CypA does not have a dominant negative effect on human CypA in dually transduced cells. Huh7.5-shRNA CypA cells non-transduced or transduced with a 3x-FLAG-tagged human CypA, mouse CypA (expressing eGFP), or both (**A**) were infected with Jc1-Gluc (MOI = 0.1). (**B**) At five dpi, cells were stained with antibodies against FLAG and NS5A for flow cytometry analysis. The percentage of NS5A+ cells was determined from the subset of cells that were FLAG+ for the samples singly transduced with the human CypA construct, eGFP +for the samples singly transduced with the mouse CypA construct or FLAG+/eGFP+ for the cells dually transduced with both the human and mouse CypA constructs. In the latter case, cells with high dual transduction were gated on as shown in (**A**) and the percentage of NS5A + cells determined from this subset. Data shown represent three independent experiments, each performed in triplicate. Two of the data points for the dually transduced, non-infected cells were zero and thus could not be plotted on a log axis. Lines and error bars represent the mean ± SD. Two-way ANOVA with Sidak multiple comparisons test used for statistical analysis. **, p<0.01; ****, p<0.0001; ns, not significant.

As we observed a lower rescue efficiency of murine compared to human CypA in the Huh7.5-shRNA CypA cells, we considered the possibility that murine CypA may have a dominant negative effect on viral replication versus simply being incompatible. To test this, Huh7.5-shRNA CypA cells were dually transduced with the bicistronic mouse CypA lentivirus expressing eGFP used above and a monocistronic lentivirus containing a C-terminally triple FLAG-tagged hCypA (Figure 4a, Figure 4—figure supplement 1). The expression of the latter, with the expected shift in size, was confirmed via western blot (Supplementary file 1). These dually transduced cells, along with singly transduced controls, were then infected with Jc1-Gluc at an MOI of 0.1 and assessed five dpi by NS5A staining (Figure 4b). We gated on cells that were highly dually positive for both mouse and human CypA and examined in this gate the fraction of HCV NS5A antigen-bearing cells. The presence of elevated human CypA along with murine CypA in the same cells did not result in a significant decline in infection, indicating no dominant negative effect.

**Figure 4—figure supplement 1.**
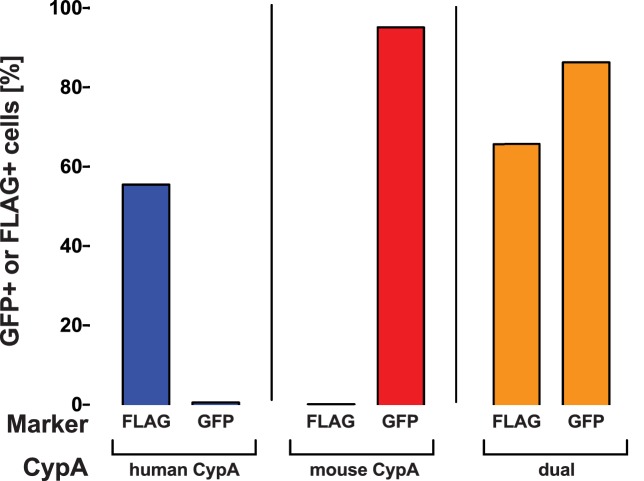
Transduction efficiency of mouse CypA-IRES-GUN and human CypA-3x FLAG in Huh7.5-shRNA CypA cells. Cells were transduced with either mouse CypA-IRES-eGFP alone, human CypA-3x FLAG, both human and mouse, or nothing for the competition experiment shown in Figure 4. Transduction efficiency of both GFP and FLAG were assessed in these lines via flow cytometry and representative data are summarized here from one set of transductions (**A**). Cell lysates were collected from Huh7.5-shRNA CypA cells transduced with these various constructs and expression determined by western blot (please see Supplementary file 1). Quantification of CypA expression was determined by taking the signal from the CypA band divided by the signal from the β-actin band and then this value divided by that from the untransduced Huh7.5-shRNA CypA cells to give the relative expression (**B**). Signal intensity was determined using LI-COR Image Studio Software (version 4.0) with background detection set to ‘Lane.’ The ‘a’ and ‘b’ above each bar delineate the different pairs of antibodies used to assess CypA expression. For ‘a,’ the signal was quantified from rabbit anti-CypA and mouse anti-β-actin; for ‘b,’ the signal was quantified from mouse anti CypA and rabbit anti- β-actin.

### Triply humanized murine CypA supports HCV spread and release of infectious particles as efficiently as human CypA

**Figure 5.**
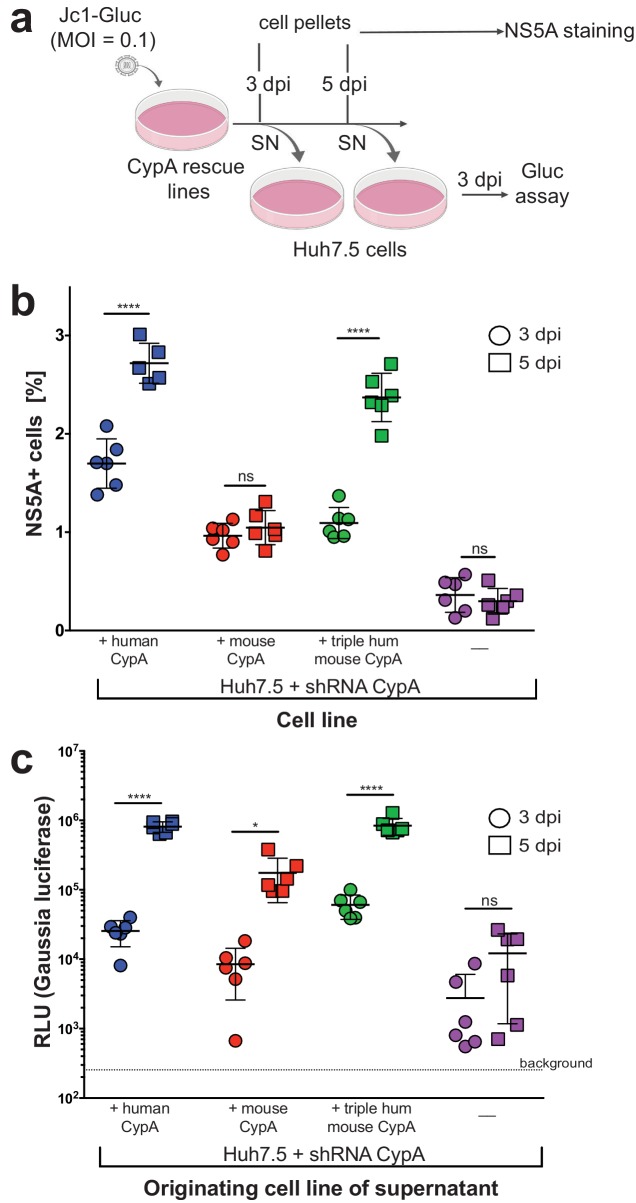
Viral spread and infectious particle production observed over time in HCV-infected rescue lines. (**A**) Schematic of experimental workflow. SN, supernatant; Gluc, *Gaussia* luciferase. Image created with BioRender. (**B**) Huh7.5-shRNA CypA cells non-transduced or transduced with human, mouse or triply humanized mouse CypA were infected with Jc1-Gluc (MOI = 0.1). At three and five dpi, as represented by circles and squares, respectively, the percentage of NS5A cells compared to naïve cells was assessed by flow cytometry. Note that one human sample had too few cells, so the NS5A staining is shown for only five, instead of six, samples. (**C**) Supernatants were collected from the infected and naïve cultures at three and five dpi and used to infect naïve Huh7.5 cells. From these infected Huh7.5 cells, supernatants were then collected at three dpi and luciferase activity once more assessed. Circles and squares indicate, respectively, the supernatants collected at 3 and 5 dpi following the infection for which NS5A staining is shown in (**B**). Results shown are from two representative experiments, each with triplicate samples. Lines and error bars represent the mean ± SD. Ordinary two-way ANOVA test performed followed by Sidak’s multiple comparison test, with the mean value for each cell line at three dpi compared to its mean at five dpi. *, p<0.05; ****, p<0.0001, ns, not significant.

Although we readily observed replication of the Jc1-Gluc genome in our rescue lines by our luminometry readout, we also tested whether this replication was occurring in only the subset of cells initially infected by the inoculum or spreading across the culture over time (Figure 5a). We took the parental Huh7.5-shRNA CypA cells plus the three rescue lines that displayed replication (mouse, human, or triply humanized mouse CypA) and infected them with Jc1-Gluc at an MOI of 0.1. At three and five dpi, viral spread was assessed by NS5A staining (Figure 5b), which significantly increased over time only for the +human CypA and +triply humanized mouse CypA cultures. Although significantly less compared to these two lines, the number of NS5A positive cells in the mouse rescue line was still significantly higher than that of the non-transduced Huh7.5-shRNA CypA cells. As the NS5A staining is less sensitive compared to the luminometry assay, we wanted to further confirm that infectious particle production was occurring and thus contributing to viral spread. Supernatants from the infected CypA rescue lines were also collected at three and five dpi, applied to naïve Huh7.5 cells and replication assessed three dpi by luminometry (Figure 5c). As expected, the supernatants collected three dpi from all rescue lines resulted in lower replication in Huh7.5 cells compared to the five dpi supernatants, indicating an increase in infectious particle production over time. Supernatants collected from parental Huh7.5 shRNA CypA cells did not exhibit an increase in infectious particles over time, as replication levels in Huh7.5 cells did not significantly increase following infection with the five dpi supernatant. However, there clearly was still some infectious particle production occurring as the level of replication was at least a log above background.

### Characterizing HCV replication in an engineered murine hepatoma line

**Figure 6.**
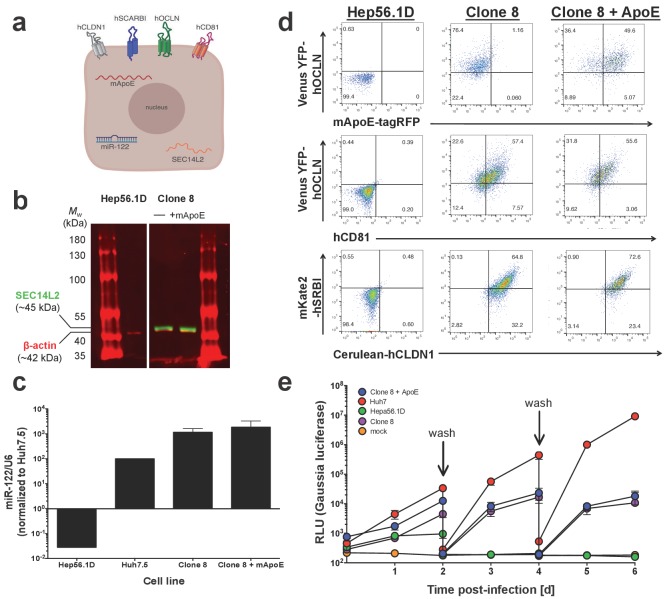
Clone 8 cells express multiple factors important for the HCV life cycle. (**A**) Schematic of the engineered murine hepatoma cell line, Clone 8 + murine ApoE (mApoE) derived from Hep56.1D cells. (**B**) SEC14L2 expression verified by western blot. Mouse anti-human SEC14L2 shown in green (~45 kDa) and rabbit anti-β actin shown in red (~42 kDa). (**C**) miR-122 expression was assessed by RT-qPCR. Results are normalized to U6 and shown as fold change relative to Huh7.5 cells (set at 100). (**D**) Clone 8 ± ApoE express human CD81, Venus YFP-human OCLN, Cerulean-human CLDN1, mKate2-human SRBI and murine ApoE-tagRFP as assessed by flow cytometry. (**E**) The kinetics of HCV replication were assessed in Clone 8 ± ApoE, the parental Hep56.1D cells and the highly permissive human hepatoma cell line Huh7. Cells were infected with Jc1-Gluc at an MOI of 0.1 and supernatants collected daily for six days. On even-numbered days as indicated by the labeled arrows, cells were washed with PBS and the media changed to assess de novo replication. Lines and error bars represent the mean ± SD.

We next moved into a murine context to see how overexpression of murine CypA, human CypA or our triply humanized/murinized mutants might impact HCV replication. To this end, we generated murine Hep56.1D hepatoma cells expressing via lentiviral transduction a variety of factors already established as important to the HCV life cycle: the four HCV human entry factors discussed above (OCLN, CLDN1, SCARBI, and CD81); miR-122 to aid in replication; and SEC14L2, which is absent in hepatoma cells but well-expressed in primary human hepatocytes and has allowed for the in vitro replication and low level viral particle production of normally non-permissive genotypes and clinical isolates of HCV (Saeed et al., 2015). This line, termed Clone 8, we also transduced with murine ApoE (mApoE), which as described above serves in viral packaging and release (Frentzen et al., 2014; Long et al., 2011), to form Clone 8 + ApoE cells (Figure 6a). Expression of all these factors was verified by a combination of flow cytometry, western blot and RT-qPCR (Figure 6b–d) and the replicative kinetics of Jc1-Gluc assessed over six days, with Huh7 cells serving as a positive control (Figure 6e). Replication was consistently highest in the Huh7 cells, with the difference between the Clone 8/Clone 8 + ApoE and Huh7 cells increasing over time till by six dpi there was an approximately three-log difference in luciferase activity. The levels of replication in the parental Hep56.1D cells traced that of mock, with no de novo replication observed after the first wash of cells at two dpi. The addition of mApoE to the Clone 8 cells did not have an impact on the replication kinetics.

**Figure 7.**
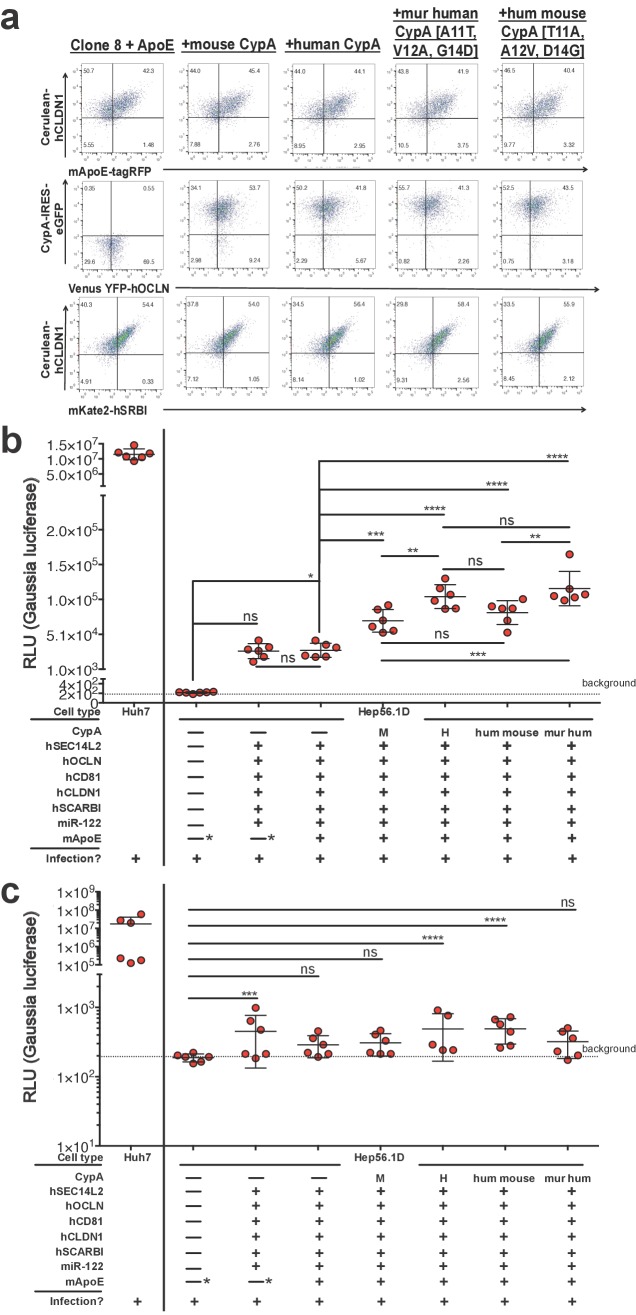
Expressing CypA variants in Clone 8 ± ApoE cells increases HCV replication. (**A**) Clone 8 + ApoE cells were transduced with mouse, human, triply murinized human, or triply humanized mouse CypA in bicistronic lentiviral constructs expressing eGFP. Transduction efficiency assessed by flow cytometry. (**B**) The transduced Clone 8 cells were infected with Jc1-Gluc (MOI = 0.1) and the media changed daily. The different factors transduced into the Hep56.1D cells are specified below the graph. Note that the "—*” for mApoE indicates that these cells were not transduced to express exogenous mApoE; their endogenous expression was not disrupted. Results shown are from two representative experiments, each with triplicate samples. *Gaussia* luciferase activity was assessed in these supernatants as a proxy for HCV replication and is expressed as relative luminescence units (RLU). Ordinary one-way ANOVA test performed on the Hep56.1D and derivative lines followed by Tukey’s multiple comparisons test with a single pooled variance. The dashed line labeled ‘background’ is the average signal from supernatants of naïve cells. (**C**) As in (**B**), Huh7 cells, Hep56.1D and derivative lines as shown on the x-axis were infected with Jc1-Gluc (MOI = 0.1) and supernatants collected at six dpi to infect naïve Huh7.5 cells. Following infection of these Huh7.5 cells with the supernatants, the media was changed daily. Shown is the *Gaussia* luciferase activity of the Huh7.5 supernatants three dpi with the origin of the supernatants used for the original infection indicated on the X axis. Lines and error bars represent the mean ± SD. The dashed line labeled ‘background’ is the average signal from supernatants of naïve cells. Ordinary two-way ANOVA test performed on the Hep56.1D and derivative lines followed by Dunnett’s multiple comparison test, with all means compared to that of Hep56.1D. Lines and error bars represent the mean ± SD. *, p<0.05; **, p<0.01; ***, p<0.001; ****, p<0.0001.

Human, mouse, and the triply humanized/murinized CypA constructs were then transduced into the Clone 8 + ApoE cells (Figure 7a) and the replication assessed once more by luminometry following Jc1-Gluc infection (Figure 7b). At six dpi, supernatants from Hep56.1D cells showed negligible luciferase activity as expected. The Clone 8 and Clone 8 + ApoE cells reached similar levels to one another, both about two logs higher than the parental line. The addition of any of the CypA constructs tested resulted in a significant increase in luciferase activity, with human or triply murinized human CypA the greatest, albeit by a small but significant margin.

Based off these differences in replication, we then assessed whether there was any difference in infectious particle production. Supernatants from infected Hep56.1D and the Clone 8 lines were collected six dpi and used to infect naïve Huh7.5 cells where replication was then assessed three dpi by luminometry (Figure 7c). While the supernatants collected from Clone 8, Clone 8 + ApoE + human CypA and Clone 8 + ApoE + triply humanized mouse CypA cells did result in significantly higher replication in Huh7.5 cells compared to the Hep56.1D supernatants, the increase remained on average less than a log.

## Discussion

Although advances in available therapies, even cures, for HCV are striking, preventive measures, such as a vaccine, have still not been developed. In the United States alone over the past decade, there has been a more than 133% increase in acute HCV incidence, which is strongly associated with the domestic opioid epidemic (Zibbell et al., 2018; Zibbell et al., 2014; Zibbell et al., 2015). Even with treatment, which has its own financial and logistical limitations, reinfection can still occur and is likely among high-risk individuals such as injection drug users and HIV-positive men who have sex with men (Falade-Nwulia and Sulkowski, 2017; Hill et al., 2017; Salazar-Vizcaya et al., 2018). Thus, HCV research is still greatly needed and immunocompetent models necessary more than ever for vaccine development.

Due to the limited host range of HCV, research has focused on finding an immunocompetent small animal model without the financial and ethical restrictions associated with chimpanzees. At the heart of such research is understanding the basic virology and host restrictions of HCV, chipping away at the known barriers to the viral life cycle in a murine context such as at the level of entry (Ding et al., 2017; Dorner et al., 2011; Ploss et al., 2009) and viral particle assembly and release (Frentzen et al., 2014; Long et al., 2011; Vogt et al., 2013). Viral replication in murine cells has consistently been low, and increasing replication has relied on selectable subgenomic replicons (Long et al., 2011) or disrupting cell-intrinsic antiviral responses (Aly et al., 2011; Anggakusuma et al., 2015; Frentzen et al., 2014; Long et al., 2011; Nandakumar et al., 2013; Vogt et al., 2013). Similarly, to observe even low levels of HCV replication in vivo using transgenic mouse models, blunting of innate immune signaling is also needed (Dorner et al., 2013; Vogt et al., 2013). Although the entire HCV life cycle can be completed with robust replication in xenorecipient mice engrafted with human or stem cell-derived hepatocytes (Bissig et al., 2010; de Jong et al., 2014; Kosaka et al., 2013; Mercer et al., 2001; Meuleman et al., 2005; Tesfaye et al., 2013; Washburn et al., 2011), the immunocompromised nature of these mice precludes studying immune responses.

It may not be possible to completely close this gap between levels of HCV replication in human versus other small animal model hepatocytes, but we sought to determine how species-specific differences in CypA – one of the few host factors experimentally validated as essential for viral replication – might contribute to these observed differences. We argue that such work has an impact on the development of an immunocompetent small animal model for studying HCV infection, which remains a critical need.

In the present study, we assessed the ability of CypA orthologs from a variety of species to support HCV replication in a human context. Besides chimpanzee, bonobo, gorilla, olive baboon and rhesus macaque, which are 100% identical to human CypA at the amino acid level, all the remaining orthologs tested varied from hCypA by no more than six amino acids. Recent work has shown that single nucleotide polymorphisms in the hCypA gene can abrogate HCV infection in primary human hepatocytes (von Hahn et al., 2012). The ability of these single missense mutations in the hCypA coding sequence to dramatically reduce HCV replication lent credence to the possibility that the small number of amino acid differences that differentiate hCypA from the orthologs we tested might have important functional significance in the context of HCV replication. In our system, the only other unique CypA ortholog able to rescue HCV replication during infection with Jc1-Gluc to the same extent as human was orangutan, which differs by only one amino acid.

Tree shrew, pigtailed macaque and squirrel monkey CypA did not significantly increase replication compared to the non-transduced Huh7.5-shRNA CypA cells. Although we were not able to robustly detect squirrel monkey CypA and observed no signal for the pigtailed macaque TRIM5-CypA fusion protein by western blot, these two orthologs could still be expressed as commercial antibodies with confirmed specificity for these two orthologs are not available. This is especially pertinent as the antibodies we used had listed specificity for human and/or mouse. Compared to CypA from these two species, the pigtailed macaque and squirrel monkey orthologs differed by multiple residues that could impact antibody binding. Tree shrew CypA was readily detected with either of the two antibodies tested, so its failure to rescue HCV replication is more conclusive. It is important to underscore that our observations for tree shrew (and for squirrel monkey and pigtailed macaque if they are in fact expressed) do not mean that this species could not support HCV replication if its CypA ortholog was expressed in its native cellular environment. The amino acid differences in CypA might be best suited for interaction with species-specific factors for which the human ortholog is not an apt replacement. Indeed, recent work has demonstrated that HCV can replicate efficiently in pigtailed macaque stem cell-derived hepatocyte-like cells after overcoming the CD81- and OCLN-related barriers to viral entry (Sourisseau et al., 2013), although whether the TRIM5-CypA fusion tested here was expressed in these particular cells is unknown. Likewise, rhesus macaque primary hepatocytes were also shown to support HCV infection in vitro and in vivo, albeit only at low levels (Scull et al., 2015). Of course, it is still possible that a given CypA ortholog, even in its native context, would fail to properly interact directly with the necessary HCV proteins to facilitate replication. For example, the T73I difference between human and squirrel monkey CypA (if indeed expressed) could have a meaningful impact on such interactions as it is adjacent to G72, one of the CsA-binding residues.

In contrast, murine CypA was the only other ortholog that demonstrated some ability to rescue HCV replication. This was especially striking as mouse was the most evolutionarily distant species we examined and the murine CypA ortholog had the most differences in amino acid sequence compared to human. As we tested, this was not due to a dominant negative effect of murine CypA on replication as HCV could still replicate efficiently in the presence of both human and murine CypA. Thus, we created a series of mutants where we interchanged the six differing residues between human and mouse CypA. We were able to confirm expression of these mutants by western blot with at least one of the antibodies we tested. We cannot adequately control for the varying antibody reactivity, so comparing the expression of different CypA constructs with each other is difficult as lower *detected* expression could indicate lower antibody reactivity. This is highlighted by the differences between the two antibodies we tested, with one indicating a more than 100-fold higher expression for a CypA variant compared to the non-transduced Huh7.5-shRNA CypA cells while the other antibody reached only 20-fold. Since we could detect all of the CypA mutants by western blot, expressed them all in the same backbone construct, and observed high transduction efficiency, conceivably all the transduced cells would be able to express at least some level of the CypA variant.

Of the six amino acids differing between the two orthologs, single ‘humanization’ of mCypA residues 12, 14, 52, and 76 had the greatest impact, resulting in increased rescue. Conversely, ‘murinization’ of hCypA residues led to significant decreases in rescue ability across all residues tested. Strikingly, simultaneously altering the three residues clustered together that differed between human and mouse (positions 11, 12 and 14) resulted in a gain-of-function phenotype for murine CypA and a loss-of-function phenotype for human CypA. The gain-of-function phenotype was not further exacerbated in the triply humanized murine CypA by introducing the S52C mutation as well, which as a single mutant had shown rescue efficiency similar to the individual mutants at positions 12 and 14. None of the differences between human and mouse CypA, or indeed for any of the CypA orthologs, overlapped with residues of the active site/CsA-binding site of CypA, which as further described below has been characterized as important for interaction with HCV proteins. This raises the intriguing possibility that this cluster of three residues plays a role in maintaining these interactions with the HCV replication machinery and/or associating with host factors that help facilitate such interactions. The location of the three residues on the face of the protein opposite the CsA-binding/active site makes it unclear whether changing these residues leads to a loss of PPIase activity that would explain the accompanying change in phenotype. Identifying these potential conformational changes and/or host factors would provide novel insights into the still unclear mechanism of action by which CypA promotes HCV replication.

Furthermore, we tested in our system the impact of mouse, human or triply humanized mouse CypA on infectious particle production and viral spread over time. Even though spread over time was not detectable by NS5A staining in the murine CypA rescue cells, the more sensitive luminometry assay still demonstrated infectious particles were being released into the supernatant. Thus, in our system, the HCV life cycle is still being completed and the virus able to propagate beyond the cells initially infected with the inoculum.

Although informative, these findings were all in the context of a human cell and thus whether or not this ‘humanization’ of mCypA would have the same effect in murine cells was unclear. In the same vein, it was not known if exogenous expression of human CypA in a murine context would boost HCV replication. As briefly alluded to above, there have been numerous efforts to engineer murine cells for studying HCV. Previous efforts using subgenomic replicons indicated replication could occur in murine embryonic fibroblasts (MEFs) at least to some extent and could be enhanced with the addition of miR-122 (Jopling et al., 2005; Lin et al., 2010) and deletion of IRF3 (Lin et al., 2010). Hep56.1D cells also support replication of subgenomic replicons and infectious particle release was achieved by using a selectable replicon with HCV core, E1, E2, p7 and NS2 provided in trans (Long et al., 2011). The low levels of infectious particle release initially observed could be further increased by expressing either murine or human ApoE (endogenous expression in Hep56.1D cells is low). However, in this same study, replication of transfected full-length genomes (selectable or non-selectable) was poor. Replication of subgenomic replicons was also enhanced in murine liver tumor (MLT) cells with the addition of miR-122 and disruption of IFN receptors (Anggakusuma et al., 2015) or in MEFs with blunted innate immune responses (Nandakumar et al., 2013). Similarly, replication of full-length HCV RNA following transfection in primary murine hepatocytes remained low unless MAVS, IRF1 or IFNAR — all important players in cell-intrinsic antiviral responses — were knocked out (Aly et al., 2011; Nandakumar et al., 2013). Transfecting full-length genomes into MAVS^-/-^ MLT cells expressing miR-122 and the human HCV entry factors resulted in robust replication and infectious particle production; however, in performing infections, replication declined and infectious particle release fell below background levels (Frentzen et al., 2014). The only instance of viral infection in murine cells where strong replication and particle production were demonstrated relied on the use of a blasticidin-selectable genome (Jc1-bsd) in immortalized Stat1 knockout MEFs (iMEFs) expressing the human HCV entry factors, miR-122 and mApoE (Vogt et al., 2013).

Unlike these previous efforts, we wanted to focus on the replication of a non-selectable, full-length genome during infection without exogenous disruption of cellular immune responses as this is most immediately relevant and critical for generating a successful HCV animal model. Towards this aim, we engineered murine Hep56.1D hepatoma cells to express a variety of factors to overcome the various blocks at the level of entry and replication. These so-called Clone 8 cells demonstrated sustained de novo replication compared to the parental Hep56.1D cells, regardless of whether mApoE was present. Addition of murine CypA resulted in a further increase in replication that was even greater for human CypA. The phenotypes of the triple mutants in this murine context were the reverse of those observed in Huh7.5-shRNA CypA cells, suggesting that the mechanism by which these mutants function could depend to some extent on the species specificity of the cellular environment, which will be an interesting area for future study.

In spite of the elevated replication and presence of mApoE in our Clone 8 cells, infectious particle production was not even a log greater than the parental Hep56.1D cells. This is similar to previous observations that when infecting humanized, MAVS^-/-^ MLTs with full-length genomes, infectious particle release is negligible and viral replication lower compared to transfecting in the genome (Frentzen et al., 2014). This raises the question of whether viral replication must be enhanced further in order to achieve greater viral release and/or packaging or whether factors directly important to these final steps of the viral life cycle are still missing. The evidence that infection with selectable HCVcc, so that the population is made up of cells containing HCV genome (Vogt et al., 2013), results in efficient particle release as does transfecting in RNA to ‘hit’ more cells (Frentzen et al., 2014) suggests the former. However, in both of these cases, it is unclear how much blunting the innate immune response additionally contributed to these observations. Furthermore, if there is (a) murine factor(s) that is having an inhibitory effect on particle release, as observed for murine tetherin’s block of HIV-1 particle release due to its resistance to Vpu-mediated degradation (McNatt et al., 2009), more viral replication may be required to overcome this. The luciferase assay we used is extremely sensitive, capturing even low levels of replication that may simply be insufficient for robust infectious particle production. To enhance replication, other (co-)factors/adaptors may be necessary to facilitate the interactions of CypA and are simply missing in murine cells or are incompatible orthologs of such factors. As observed for PI4KA, it is also possible that the effects of CypA we observe in the hepatoma lines tested may differ in a primary hepatocyte context and/or with the use of patient HCV isolates (Harak et al., 2017).

Understanding the mechanistic basis for the differences we observed in the respective rescue abilities of the CypA orthologs and mutants we tested remains an important area for future research. Indeed, these mutants may prove useful for delineating both the direct and indirect interactions necessary for robust HCV replication. Extensive study of human CypA has determined interactions with multiple HCV proteins. A connection between CypA and the HCV RdRp is evidenced by mutations in NS5B linked to CsA resistance (Fernandes et al., 2007; Robida et al., 2007; Yang et al., 2008a) and indications that NS5B can bind at the CypA active site (Chatterji et al., 2009; Yang et al., 2008a), which might enhance the viral protein's RNA-binding capcacity as a result (Nag et al., 2012).

The interaction of CypA with the HCV phosphoprotein NS5A is far better characterized. Mutations specifically in domain 2 of NS5A were linked to CsA treatment resistance among patients (Goto et al., 2009). CypA stably binds NS5A and this interaction is dependent on the protein’s PPIase activity (Chatterji et al., 2010b), which promotes the isomerization of multiple conserved proline residues in domains II and III of NS5A, contributing to the protein’s proper folding (Coelmont et al., 2009; Foster et al., 2011; Grisé et al., 2012; Hanoulle et al., 2009; Verdegem et al., 2011). As with NS5B, CypA is also believed to promote the RNA-binding capacity of NS5A (Nag et al., 2012). Upon inhibition of CypA activity, lipid and protein trafficking patterns associated with the formation of lipid droplets and the VLDL synthesis pathway are altered (Anderson et al., 2011). This has led to the hypothesis that CypA might facilitate the trafficking and assembly function of NS5A, which is known to localize to lipid droplets during the assembly of progeny virions (Nag et al., 2012). Furthermore, whether CypA expression or just its PPIase activity is inhibited, formation of double membrane vesicles (DMVs) – a prevalent component of the membranous web derived from the host endoplasmic reticulum (ER) which serves as scaffolding for viral genome replication (Paul et al., 2013; Romero-Brey et al., 2012) – is abrogated (Chatterji et al., 2015). A portion of host CypA is already localized to ER compartments, which could be advantageous for later HCV replication complex formation in these same regions (Chatterji et al., 2010a). Within these DMVs, HCV replication complexes can then more safely assemble outside of the purview of host nucleic acid sensors and other defenses. Indeed, treatment of infected cells with cyclophilin inhibitors leads to changes in the ER that temporarily prevent re-infection (Chatterji et al., 2016). Finally, an interaction between CypA and the viral assembly factor and protease NS2 has also been suggested, perhaps impacting the cleavage of NS2 from NS3 by the NS2-NS3 protease (Ciesek et al., 2009) and/or the overall proteolytic processing of the HCV polyprotein (Kaul et al., 2009). It will be important to determine if the mutants and orthologs we examined here maintain or disrupt these known interactions or instead participate in novel, undocumented interactions.

In this study, we focused on the contribution of CypA to the host restriction of HCV, but undertaking these same experiments with the other well-described HCV replication factor PI4KA is also of great interest. The percent amino acid similarity between human PI4KA and its orthologs in the species we examined in this study is lower compared to CypA, ranging from 83.8% (chimpanzee) to 99.5% (rhesus macaque) for PI4KA (Supplementary file 2) versus 97–100% for CypA. However, assessing the PI4KA orthologs for all of these species is challenging, as a strong candidate for a PI4KA ortholog is either absent for a given species (i.e. tree shrew) or is a possible ortholog by our own sequence homology search but not formally annotated (i.e. gorilla and bonobo). Some annotations for a predicted PI4KA ortholog are also more than 100 amino acids shorter than that of human, which is 2102 amino acids. Thus, determining the ‘true’ PI4KA ortholog for a given species requires additional corroboration.

The engineered murine hepatoma line we have generated will serve as a ready platform for testing the potential of additional human factors or humanized murine factors to increase the replication capacity of HCV in these cells. If augmentation of viral replication is seen in vitro, genetic engineering approaches could be utilized to generate mice expressing these human orthologs or ‘humanized’ alleles . Such genetic humanization approaches would finally create the possibility to study HCV infection and pathogenesis in an immunocompetent small animal model, informing future vaccine development and testing.

## Materials and methods

**Key resources table:** 

Reagent type (species) or resource	Designation	Source or reference	Identifiers	Additional information
antibody	mouse monoclonal anti-B-actin	Cell Signaling Technology	Cell Signaling Technology:3700	used at 1:1000 for western blot
antibody	rabbit polyclonal anti-CypA	Cell Signaling Technology	Cell Signaling Technology:2175S	used at 1:1000 for western blot
antibody	rabbit monoclonal anti-GFP (D5.1) XP	Cell Signaling Technology	Cell Signaling Technology:2956S	used at 1:1000 for western blot
antibody	goat anti-mouse IgG (H + L) secondary, Dylight 680	Thermo Fisher Scientific	Thermo Fisher Scientific:35568	used at 1:10,000 for western blot
antibody	goat anti-mouse IgG (H + L) secondary, Dylight 800	Thermo Fisher Scientific	Thermo Fisher Scientific:SA535521	used at 1:10,000 for western blot
antibody	goat anti-rabbit IgG (H + L) cross-adsorbed secondary, Alexa 700	Invitrogen	Invitrogen:A-21038	used at 1:250 for flow cytometry
antibody	goat anti-mouse IgG (H + L) cross-adsorbed secondary, Alexa 647	Invitrogen	Invitrogen:A-21235	used at 1:250 for flow cytometry
antibody	rabbit anti-β actin	Cell Signaling Technology	Cell Signaling Technology:4970S	used at 1:2000 for western blot
antibody	mouse monoclonal anti-human CypA	AbCam	AbCam:58144	used at 1:1000 (from 1 mg/mL stock) for western blot
antibody	monoclonal, human CD81 conjugated to PE	BD Biosciences, Inc.	BD Biosciences, Inc:BDB555676	used at 1:200 for flow cytometry
antibody	mouse monoclonal anti-human SEC14L2	LifeSpan BioSciences, Inc.	LifeSpan BioSciences, Inc:LS-B11733	used at 1:1000 for western blot
antibody	rabbit monoclonal anti-FLAG	Cell Signaling Technology	Cell Signaling Technology:14793S	used at 1:1500 for flow cytometry
antibody	mouse monoclonal 9E10	Charles Rice (Rockefeller University)		antibody against HCV NS5A; published in Lindenbach et al., 2005
cell line (Homo sapiens)	Huh7.5.1	Frank Chisari (The Scripps Research Institute)	RRID:CVCL_E049	Human hepatoma cell line
cell line (Mus musculus)	Hep56.1D	CLS Cell Lines Service GmbH (Eppelheim, Germany)	RRID:CVCL_5769	Murine hepatoma cell line
cell line (Mus musculus)	Clone 8	this paper		Please see methods section in manuscript for detailed information concerning the generation of this line from Hep56.1D cells and the plasmids used
cell line (Homo sapiens)	293T	Charles Rice (Rockefeller University)	RRID:CVCL_0063	Human embryonic kidney cell line
cell line (Homo sapiens)	Huh7.5	Charles Rice (Rockefeller University)	RRID:CVCL_7927	Human hepatoma cell line
cell line (Homo sapiens)	Huh7.5-shRNA-irrel	von Hahn et al., 2012		Human hepatoma cell line Huh7.5 expressing an shRNA against an irrelevant target
cell line (Homo sapiens)	Huh7.5-shRNA-CypA	von Hahn et al., 2012		Human hepatoma cell line Huh7.5 expressing an shRNA against human CypA
cell line (Homo sapiens)	Huh7	Charles Rice (Rockefeller University)	RRID:CVCL_0336	Human hepatoma cell line
commercial assay or kit	Gibson Assembly kit	New England Biolabs	NEB:E5510s	
commercial assay or kit	In-Fusion HD Cloning	Clontech	Clontech:639647	
commercial assay or kit	QuikChange XL Site-Directed Mutagenesis kit	Agilent	Agilent:200517	
commercial assay or kit	QuikChange Multi Site-Directed Mutagenesis kit	Agilent	Agilent:200514	
commercial assay or kit	Luc-Pair Renilla Luciferase HS Assay Kit	GeneCopoeia	GeneCopoeia:LF012	
commercial assay or kit	T7 RiboMAX Express Large Scale RNA Production kit	Promega	Promega:PRP1320	
recombinant DNA reagent (plasmid)	Jc1(p7nsGluc2a)	Marukian et al., 2008		
recombinant DNA reagent (plasmid)	pWPI-human CypA-IRES-GUN	von Hahn et al., 2012		plasmid expressing human CypA open reading frame followed by IRES-regulated green fluorescent protein (GFP)-ubiquitin-neomycin (GUN) fusion protein
recombinant DNA reagent (plasmid)	pWPI-orangutan CypA-IRES-GUN	this paper		plasmid expressing orangutan CypA open reading frame followed by IRES-GUN
recombinant DNA reagent (plasmid)	pWPI-tree shrew CypA-IRES-GUN	this paper		plasmid expressing tree shrew CypA open reading frame followed by IRES-GUN
recombinant DNA reagent (plasmid)	pWPI-squirrel monkey CypA-IRES-GUN	this paper		plasmid expressing squirrel monkey CypA open reading frame followed by IRES-GUN
recombinant DNA reagent (plasmid)	pWPI-mouse CypA-IRES-GUN	this paper		plasmid expressing pigtailed macaque TRIM-CypA open reading frame followed by IRES-GUN
recombinant DNA reagent (plasmid)	pWPI-humanized mouse CypA-IRES-GUN mutants	this paper		The six amino acid residues that differ between human and mouse CypA were changed one at a time or in varying combinations in mouse CypA to make it ‘humanized.’ Please see Materials and methods section for the generation of all these plasmids
recombinant DNA reagent (plasmid)	pWPI-murinized human CypA-IRES-GUN mutants	this paper		The six amino acid residues that differ between human and mouse CypA were changed one at a time or in varying combinations in human CypA to make it ‘murinized.’ Please see Materials and methods section for the generation of all these plasmids
recombinant DNA reagent (plasmid)	pWPI-pigtailed macaque TRIM-CypA-IRES-GUN	this paper		
recombinant DNA reagent (plasmid)	pLVX-IRES-puro	Clontech	Clontech:632183	
sequence-based reagent	all primers beginning with PU-O-	Integrated DNA Technologies		please see Table 1 in the Materials and methods section for all primer sequences and their use
software, algorithm	GraphPad Prism	GraphPad Software, Inc	Version 6.0e	
software, algorithm	FlowJo	FlowJo, LLC	Version 10.4.2	
software, algorithm	MacVector	MacVector, Inc	Version 12.7.4	

### Cell lines and culture conditions

Huh7.5, Huh7 and 293 T cells were generously provided by Charles Rice (Rockefeller University, NY) and Huh7.5.1 cells as a kind gift from Frank Chisari (The Scripps Research Institute, CA). Huh7.5 cells expressing a shRNA against either human CypA (Huh7.5-shRNA CypA) or an irrelevant target (Huh7.5-shRNA irrel) were graciously provided by Thomas von Hahn and Sandra Ciesek (Hannover Medical School, Germany; University of Duisburg-Essen, Germany) (von Hahn et al., 2012). Huh7 cells were obtained from the American Tissue Culture Collection (ATCC) and Hep56.1D cells from CLS Cell Lines Service GmbH (Eppelheim, Germany). The Hep56.1D-derived "Clone 8" cells were generated as described below. All cells have been authenticated and are clear of mycoplasma contamination. All cell lines were maintained in Dulbecco’s modified Eagle medium (DMEM) (Thermo Fisher) supplemented with 10% (v/v) fetal bovine serum (FBS) (Omega Scientific). To select for the shRNA, Huh7.5-shRNA CypA and Huh7.5-shRNA irrel cells were maintained under blasticidin selection at 5 ug/mL (BioVision). Cells transduced with lentivirus expressing the different CypA orthologs (see below) were selected for with Geneticin (G418) (Teknova) at 750 µg/mL. All infections were performed in the absence of antibiotics. Cells were maintained at 37°C in a 5% (v/v) CO_2_, 20% (v/v) O_2_ environment.

### Antibodies

The monoclonal mouse anti-NS5A 9E10 antibody was generously provided by Charles Rice (Rockefeller University, NY). The following commercial primary antibodies were used: rabbit anti-FLAG for flow cytometry (1:1500, #14793S, Cell Signaling Technology); rabbit anti-human SEC14L2 for western blot (1:1000, catalog #LS-B11733, LifeSpan BioSciences, Inc); mouse anti-human CypA for western blot (1 µg/ul, catalog #58144, AbCam); human CD81 conjugated to PE monoclonal for flow cytometry (1:200, catalog #BDB555676, BD Biosciences), rabbit anti-β actin for western blot (1:2000, catalog #4970S, Cell Signaling Technologies), mouse anti-β actin for western blot (1:1000, catalog #3700S) and rabbit anti-human/mouse/rat/monkey CypA (catalog #2175S, Cell Signaling Technologies). The following commercial secondary antibodies were used: goat anti-mouse Alexa 647 (1:250, catalog #A-21235, Invitrogen) for flow cytometry; goat anti-rabbit Alexa 700 for flow cytometry (1:250, catalog #A-21038, Invitrogen); goat anti-mouse Dylight 800 for western blot (1:10,000, catalog #SA535521, Thermo Fisher Scientific); and goat anti-rabbit Dylight 680 for western blot (1:10,000, catalog #35568, Thermo Fisher Scientific).

### Amino acid sequence alignment

A multiple sequence alignment of the following CypA amino acid sequences was performed in MacVector (v. 12.7.4) using the ClustalW multiple sequence alignment (v1.83): human (NCBI Reference Sequence NP_066953.1), chimpanzee (NCBI Reference Sequence XP_001148412.1), bonobo (NCBI Reference Sequence XP_008967123.1), gorilla (NCBI Reference Sequence XP_018886247.1), orangutan (NCBI Reference Sequence NP_001126060.1), olive baboon (NCBI Reference Sequence XP_003896076.1), rhesus macaque (NCBI Reference Sequence NP_001027981.1), pigtailed macaque TRIM5-CypA (Genbank AGA83499.1), squirrel monkey (NCBI Reference Sequence XP_003923963.1), mouse (NCBI Reference Sequence NP_032933.1) and tree shrew (NCBI Reference Sequence XP_006166088.1).

The additional amino acid alignment for PI4KA in Supplementary file 1 was performed using ClustalW in the same manner as for CypA. Since not all species, such as bonobo and gorilla, had genes annotated as PI4KA, sequences were retrieved from Ensembl by examining orthologs specifically for the human PI4KA gene (**ENSG00000241973). I**n the case of one-to-many orthologs, the one with the highest whole genome alignment (WGA) coverage score (calculated by Ensembl) was used to access the accompanying amino acid sequence. The PI4KA ortholog gene sequences from all species used for the CypA alignment had WGA coverage scores of 99+ (maximum score = 100) except for tree shrew, which was not included in the alignment due to its poor WGA score of 72.23 and less than 70% sequence identity. Indeed, many residues listed for the putative tree shrew ortholog were ‘X.’ The accession IDs for the amino acid sequences used in the alignment are shown in Supplementary file 1.

### Plasmid construction

The bicistronic lentiviral vector pWPI-IRES-GUN expressing both the human CypA open reading frame (ORF; NCBI Reference Sequence NM_021130.4) and an IRES-regulated green fluorescent protein (GFP)-ubiquitin-neomycin resistance (GUN) fusion protein was kindly provided by Thomas von Hahn (Hannover Medical School; Germany). As human, rhesus macaque, bonobo, gorilla, olive baboon, and chimpanzee CypA were 100% identical at the amino acid level, despite variation in the nucleic acid sequence, human CypA was used as the representative for these five other species and thus served as a proxy for the functional phenotype of the other orthologs. The CDS utilized for the remaining species of interest are as follows: orangutan CypA (NCBI Reference Sequence NM_001132588.1), tree shrew CypA (NCBI Reference Sequence XM_006166026.2), mouse CypA (NCBI Reference Sequence NM_008907.1), squirrel monkey CypA (NCBI Reference Sequence XM_003923914.2), and pigtailed macaque TRIM5-CypA (GenBank Sequence JX865267.1).

The orangutan CypA ORF, which differs by one amino acid from human (F8L), was generated by PCR mutagenesis with primers PU-O-3432 and PU-O-3428 (Table 1) that simultaneously made the amplified coding region compatible for In-Fusion HD Cloning (Takara Bio). The ORFs of tree shrew, mouse, squirrel monkey, and pigtailed macaque orthologs were synthesized as gBlock gene fragments (Integrated DNA Technologies) containing overlapping regions with the pWPI vector for subsequent In-Fusion HD Cloning.

**Table 1.** Oligonucleotides utilized in constructing the described plasmids.

Primer ID	Nucleotide sequence (5’−3’)
PU-O-3432	TGCAGCCCGTAGTTTACTAGTTTATTCGAGTTGTCCACAGTCAG
PU-O-3428	ACCTGCAGGCGCGCCGGATCCATGGTCAACCCTACCGTGTTCTTGGACATT
PU-O-3853	TTCTTCGACATTACGGTCGACGGCGAGCCC
PU-O-3854	GGGCTCGCCGTCGACCGTAATGTCGAAGAA
PU-O-3855	TTCGACATTGCCGCCGACGGCGAGCCC
PU-O-3856	GGGCTCGCCGTCGGCGGCAATGTCGAA
PU-O-3857	ATTGCCGTCGACGACGAGCCCTTGGGC
PU-O-3858	GCCCAAGGGCTCGTCGTCGACGGCAAT
PU-O-3859	TATAAGGGTTCCTCCTTTCACAGAATTATTCC
PU-O-3860	GGAATAATTCTGTGAAAGGAGGAACCCTTATA
PU-O-3861	GGCACTGGTGGCAGGTCCATCTATGGG
PU-O-3862	CCCATAGATGGACCTGCCACCAGTGCC
PU-O-3863	AAGATCACCATTTCCGACTGTGGACAACTC
PU-O-3864	GAGTTGTCCACAGTCGGAAATGGTGATCTT
PU-O-3871	TTCTTCGACATCGCCGCCGATGACGAG
PU-O-3872	CTCGTCATCGGCGGCGATGTCGAAGAA
PU-O-3873	TTCGACATCACGGTCGATGACGAGCCC
PU-O-3874	GGGCTCGTCATCGACCGTGATGTCGAA
PU-O-3875	ATCACGGCCGATGGCGAGCCCTTGGGC
PU-O-3876	GCCCAAGGGCTCGCCATCGGCCGTGAT
PU-O-3877	TATAAGGGTTCCTGCTTTCACAGAATTATTCC
PU-O-3878	GGAATAATTCTGTGAAAGCAGGAACCCTTATA
PU-O-3879	GGCACTGGCGGCAAGTCCATCTACGGAGAG
PU-O-3880	CTCTCCGTAGATGGACTTGCCGCCAGTGCC
PU-O-3881	AAGATCACCATTGCTGACTGTGGACAG
PU-O-3882	CTGTCCACAGTCAGCAATGGTGATCTT
PU-O-1755	ATGAGCGGCAGAGTCGGCGATCTGA
PU-O-1756	TTATTTCGGGGTGCCTGCCCCCAGC
PU-O-1943	TATTTCCGGTGAATTCCTCGAGATGAGCGGCAGAG
PU-O-1944	GGGAGGGAGAGGGGCGGGATCCTTATTTCGGGGTG
PU-O-3851	CTTGCATGCCTGCAGGTCGACATGGTCAACCCCACC
PU-O-3852	CGGCCAGTGAATTCGAGCTCGGTACCTTATTCGAGTTGTCC
PU-O-4211	CGGCCAGTGAATTCGAGCTCGGTACCTTAGAGCTGTCCACAGTC
PU-O-3424	ACCTGCAGGCGCGCCGGATCCATGGTCAACCCCACCGTGTT
PU-O-3429	TGCAGCCCGTAGTTTACTAGTTTAGAGCTGTCCACAGTCGGAAA
PU-O-3432	TGCAGCCCGTAGTTTACTAGTTTATTCGAGTTGTCCACAGTCAG

For all constructs, the pWPI-hCypA-IRES-GUN vector was digested with *BamHI* and *SpeI* to remove the hCypA and the CypA ortholog ORFs subsequently cloned in using In-Fusion HD Cloning (Takara Bio). All plasmids were confirmed by sequencing and restriction enzyme digest.

The generation of ‘murinized’ hCypA and ‘humanized’ mCypA single mutants expressing the analogous residue at one of the six amino acid positions differentiating the two orthologs was performed using PCR site-directed mutagenesis with the QuikChange XL Site-Directed Mutagenesis kit (Agilent Technologies; Santa Clara, CA) as outlined in the user manual. For the ‘murinized’ hCypA single mutants, the following primer pairs were utilized in the PCR mutagenesis reactions: PU-O-3853 and PU-O-3854 (A11T), PU-O-3855 and PU-O-3856 (V12A), PU-O-3857 and PU-O-3858 (G14D), PU-O-3859 and PU-O-3860 (C52S), PU-O-3861 and PU-O-3862 (K76R), PU-O-3863 and PU-O-3864 (A159S) (Table 1). For the ‘humanized’ mCypA single mutants, the following primer pairs were utilized in the PCR mutagenesis reactions: PU-O-3871 and PU-O-3872 (T11A), PU-O-3873 and PU-O-3874 (A12V), PU-O-3875 and PU-O-3876 (D14G), PU-O-3877 and PU-O-3878 (S52C), PU-O-3879 and PU-O-3880 (R76K), PU-O-3881 and PU-O-3882 (S159A) (Table 1). To generate the ‘murinized’ hCypA and ‘humanized’ mCypA triple mutants expressing the analogous residues at positions 11, 12, and 14, the hCypA and mCypA residue 14 single mutants were transferred into pUC19 using primers PU-O-3851 and −3852 for human; primers PU-O-3851 and −4211 for mouse. The additional mutations at residues 11 and 12 were then introduced simultaneously using the QuikChange Multi Site-Directed Mutagenesis kit (Agilent Technologies; Santa Clara, CA) with primers PU-O-4138 and PU-O-4139 for the triply ‘murinized’ hCypA and primers PU-O-4140 and PU-O-4141 for the triply ‘humanized’ mCypA. The mutant regions were then PCR amplified from pUC-19 using primers PU-O-3424 and −3429 for mouse and PU-O-3424 and −3432 to be cloned back into pWPI. For the additional murine mutants shown in Figure 3, the S52C mutation was introduced into the single mutants at residues 11, 12 and 14 as well as the 11/12/14 triple mutant using primers PU-O-3877 and PU-O-3878 with the QuikChange XL Site-Directed Mutagenesis kit.

C-terminal 3X-FLAG-tagged hCypA was made by first generating a Gblock gene fragment (Integrated DNA Technologies) for the C-terminal 3X-FLAG coding sequences with a glycine-linker sequence and appropriate backbone sequence overlap for subsequent In-Fusion HD Cloning (Takara Bio). The hCypA ORF was PCR amplified using primers PU-O-4494 and −4495, with the stop codons in the open reading frames removed to allow for the production of the C-terminal 3X-FLAG fusion. As before, these amplified regions were inserted into the pWPI backbone digested with BamHI and SpeI using In-Fusion HD Cloning (Takara Bio).

SEC14L2 was amplified from cDNA generated from cell lysates of human fetal liver cells (HFLCs) using primers PU-O-1755 and −1756 and cloned into pShuttle-CMV. SEC14L2 was then amplified by PCR with added restriction enzyme sites (XhoI at 5’ end, BamHI 3’ end) using primers PU-O-1943 and −1944 and cloned by Gibson Assembly (New England BioLabs) into pLVX-IRES-Puro (Clontech) that had been digested with XhoI and BamHI.

### Lentivirus production and transduction

Lentiviral particles containing the various pWPI-CypA constructs were produced by Xtremegene HP DNA transfection reagent (Roche Applied Science; Indianapolis, IN)-mediated co-transfection of HEK293T cells seeded twelve hours prior to transfection (4.4E6 cells per 10 cm poly-L-lysine-coated tissue culture dish) with 4 µg of the appropriate pWPI-CypA plasmid, 4 µg of HIV gag-pol, and 0.57 µg of the G protein of vesicular stomatitis virus (VSV-G) per transfection reaction. Supernatants were harvested at 24, 48, and 72 hr post-transfection, stored at 4°C and then passed through 0.45 µm membrane filters (Millipore; Darmstadt, Germany). Polybrene (final concentration of 4 μg/mL) (Sigma-Aldrich) and HEPES (final concentration of 2 mM) (Gibco) were added to all lentiviral supernatants which were aliquoted and stored at −80°C.

All lentiviral transductions were performed via spinoculation with cells seeded at a concentration of 2E5 cells per well in a six well format 24 hr prior to transduction. Cell confluency at the time of transduction was 30–40%, and 2 mL of undiluted lentivirus was added to each well. Plates were spun at 37°C, 2 hr, 2000 rpm. Media replaced with 10% FBS DMEM 6 hr post-spinoculation.

Transduction efficiency was assessed via flow cytometry for all constructs on a BD LSRII flow cytometer (BD Biosciences) with the exception of pLVX-SEC14L2-IRES-puro (western blot was used to confirm transduction due to the lack of a fluorescent marker). All flow cytometry data was processed in FlowJo Software version 10.4.2 (FlowJo, LLC).

### Generation of HCV RNA and viral stocks

HCV RNA and subsequent viral stocks were produced as previously described (Lindenbach et al., 2005). In brief, viral RNA was produced via in vitro transcription of an XbaI-linearized Jc1(p7nsGluc2A) plasmid (Marukian et al., 2008) using the T7 RiboMAX Express Large Scale RNA Production kit (Promega) as outlined in the user manual. Viral RNA was purified using the Qiagen RNeasy Mini Kit (Qiagen) following manufacturer’s instructions, and quality control was performed by gel electrophoresis to ensure no significant RNA degradation. Viral RNA stocks were stored as 5 µg aliquots at −80°C. RNA was electroporated into Huh7.5.1 cells The pellet was resuspended in the appropriate volume of cold DPBS to achieve a concentration of 1.5E7 cells/mL. 6E6 cells were then electroporated in a 2 mm path length electroporation cuvette (BTX Harvard Apparatus; Holliston, MA) with 5 µg of viral RNA using an ECM 830 Square Wave Electroporation System (BTX) at the following settings: five pulses, 99 µs per pulse, 1.1 s pulse intervals, 860V. Following a ten-minute incubation at room temperature, the electroporated cells were seeded into p150s and maintained in 5% FBS DMEM. Media was changed one day post-electroporation, and supernatants were collected daily for six days and stored at 4°C. The pooled supernatants were passed through a 0.22 µm vacuum filter and subsequently concentrated to ~100 mL in an EMD Millipore Stirred Cell (Cole-Parmer). The TCID_50_/mL (Reed and Muench, 1938) of concentrated virus was determined after one freeze-thaw by limiting dilution assay.

### Analysis of HCV infection by NS5A staining and luminometry

HCV infections of Clone 8 cells and CypA rescue experiments were conducted in a 24 well format with 3E4 cells seeded per well 12 hr pre-infection. Infections were conducted in triplicate wells using cell-culture produced Jc1-Gluc virus produced as described above. Viral inoculum was applied for 6 hr at which time the wells were washed twice with PBS and the media changed to 10% FBS DMEM. At no time during infections were antibiotics used. For kinetics experiments, 50 µL of supernatant were taken daily from each well for up to seven days and stored in 96 well plates at −20°C. As indicated in figures, when washes were performed, supernatant was collected immediately before a single wash with PBS and immediately after the subsequent replacement with fresh medium. Viral replication was quantified by measuring the luciferase activity of the supernatant using the Luc-Pair *Renilla* Luciferase HS Assay Kit (GeneCopoeia) and a Tristar^2^ LB 942 Multimode Microplate Reader (Berthold Technologies) according to manufacturer’s instructions.

For assessing infectious particle production in supernatants, infections were performed in a 96 well format, with 6.4E3 Huh7.5 cells seeded per well 12 hr pre-infection. At the time of infection with supernatant, media was removed from the wells and replaced with 50 ul of the respective supernatant (freshly collected without any freeze-thaws). The inoculum was removed and fresh media added to the wells six hpi. Supernatants from these infected Huh7.5 cells were then collected three dpi to assess *Gaussia* luciferase activity by lumionmetry.

For NS5A staining, trypsinized cells were pelleted, fixed with 4% paraformaldehyde (PFA) (Sigma-Aldrich) and permeabilized in 0.1% (w/v) Saponin and 1% (v/v) FBS in DPBS. Pellets were subsequently incubated for 1 hr at room temperature with murine-produced Clone 9E10 primary antibody specific for NS5A (Lindenbach et al., 2005), kindly provided by Dr. Charles Rice (The Rockefeller University), diluted 1:8000 in FACS buffer (1% FBS (v/v) in DPBS). Cells were washed with DPBS and then incubated at 4°C for 30 min in the dark with goat anti-mouse Alexa 647 secondary antibody (diluted 1:250, catalog #A-21235, Invitrogen). Cells were subsequently pelleted, washed once with FACS buffer and then analyzed in FACS buffer on a BD LSRII flow cytometer (BD Biosciences). All flow cytometry data were processed in FlowJo Software version 10.4.2 (FlowJo, LLC).

### Western blot

Cell pellets were lysed for 5 min on ice in RIPA buffer (50 mM Tris, pH 7–8; 150 mM NaCl, 0.1% SDS (v/v), 0.5% sodium deoxycholate (v/v), 1% Triton X-100 (v/v)) containing protease inhibitor cocktail (Sigma-Aldrich, P-8340; 1:250 dilution). Lysates were spun down for 10 min, 12,000 rpm, 4°C. The resulting supernatant was mixed with 6X Laemmli buffer (375 mM Tris pH = 6.8, 10% SDS, 50% Glycerol, 10% beta-mercaptoEtOH, 0.03% Bromo blue) and heated for 5 min at 98°C along with PageRuler protein ladder marker (Thermo Scientific). The samples were separated on a 10% or 12% (wt/vol) SDS-polyacrylamide gel in running buffer (diluted from a 10X stock containing 30.3 g Tris, 144 g glycine, 10 g SDS in 1 L of ddiH2O) at 150V for 60 min. The resolved proteins were transferred onto a 0.2 µm nitrocellulose membrane (Bio-Rad Laboratories; Hercules, CA) in transfer buffer (10X stock containing 30.3 g Tris, 144 g Glycine, 4 g SDS in 1 L ddiH2O; diluted to 1X in ddiH2O with 20% MeOH (v/v)) for 1 hr at 18V. Membranes were blocked for at least 30 min in DPBS containing 5% milk (wt/vol), washed twice with 1X TBS containing 0.5% (v/v) Tween (TBS-T), and then incubated for 1 hr at room temperature or overnight at 4°C with primary antibodies as listed in the figure legends (see ‘Antibodies’ section above for specific product information) in TBS-T. Membranes were washed three times in TBS-T, incubated for 30 min in the dark at room temperature with the appropriate secondary antibody (goat anti-mouse secondary antibody Dylight 800, Thermo Fisher Scientific, #SA535521, diluted 1:10,000 in TBS-T; goat anti-rabbit secondary antibody Dylight 680 Thermo Fisher Scientific, #35568, diluted 1:10,000 in TBS-T) and then washed three more times in TBS-T. All membranes were visualized on an Odyssey CLx Imaging System (LI-COR Biotechnology; Lincoln, NE).

Membranes that were re-probed were stripped in Restore PLUS Western Blot Stripping Buffer (Thermo Fisher Scientific, #46430) for 15 min at room temperature, washed twice with TBS-T, and then blocked and incubated with antibody as described above. Where performed, signal intensity for bands of interest was determined using LI-COR Image Studio Software (version 4.0). Note that even if the contrast and brightness levels of a membrane image are adjusted within the software, the raw intensity values remain unchanged and were used for all quantifications.

### Generation and characterization of engineered murine hepatoma line

Hep56.1D cells were transduced with lentivirus produced from the following plasmids: pTRIP-human CD81 (Flint et al., 2006), pTRIP-Venus/YFP-human OCLN (Ploss et al., 2009), pTRIP-Cerulean/CFP-human CLDN1 (Evans et al., 2007), pTRIP-mKate human SCARBI (Vogt et al., 2013), pLVX-IRES-human SEC14L2-puro (see ‘Plasmid construction’ section above) and pTRIP-miR122 (Vogt et al., 2013). Single cell-sorting was then performed, gating on highly YFP+/mKate+ cells, which were subsequently expanded and assessed for expression of the other transduced factors by a mixture of flow cytometry (all the entry factors), western blot (SEC14L2) and RT-qPCR (miR-122). miRNA was extracted from the cells using the miRNeasy mini kit (Qiagen) following manufacturer’s directions for mature miRNA isolation. cDNA was subsequently produced using the miScript II RT kit (Qiagen) and then quantified by real-time PCR using the miScript SYBR green PCR kit all according to the manufactuer’s directions. miR-122 expression was normalized to the snRNA RNU6B using the Hs_miR-122a_1 miScript Primer Assay and Hs_RNU6-2_11 miScript Primer Assay (Qiagen; compatible for mouse and human), respectively. The final clone worked with throughout this paper was termed ‘Clone 8’. These cells were subsequently transduced with lentivirus containing pTRIP-mApoE-tagRFP (Vogt et al., 2013) and various pWPI-CypA-GUN constructs (see above) prior to experiments, with tagRFP and eGFP expression, respectively, assessed by flow cytometry.

### Modeling of ‘murinized’ human CypA

The six residues differing between human and murine CypA were substituted in the structure of human CypA (PDB ID 1CWA) (Mikol et al., 1993) using the tools AutoSub and RelabelChains from AmberUtils by William D Lees (https://github.com/williamdlees/AmberUtils) run using Python (v 2.7.10). The resultant structure was visualized and labeled in PyMOL (v 2.2.0) (Schrodinger, 2015).

### Statistical analysis

All statistical analyses, as described in figure captions, were done using GraphPad Prism software (v. 6.0).

## Data Availability

All data generated or analysed during this study are included in the manuscript and supporting files.

## Additional files

Supplementary file 1. Detection of CypA expression by western blotting. All the constructs utilized in this paper were analyzed by western blot across two membranes. Shown here are the membranes after exposure to different combinations of antibodies, with the column on the left all images of one membrane and the column on the right images of the second. Whether the bands observed in a given set of membranes originated from the goat, mouse or both secondary antibodies is delineated at the left of each row. The proteins represented by the bands are also labeled, with ‘(M)’ and ‘(R)’ indicating signal from anti-mouse or anti-rabbit secondary, respectively. The blots shown in (A) were incubated with the following primary antibodies: rabbit anti-GFP (1:1000, Cell Signaling Technologies #2956S), mouse anti-β-actin (1:1000, Cell Signaling Technologies, #3700), and rabbit anti-CypA (1:1000, Cell Signaling Technologies #2175S). These blots were stripped and re-probed in (B) with the following primary antibodies: rabbit anti-GFP (1:1000, Cell Signaling Technolgoies #2956S), rabbit anti-β-actin (1:1000, 4970S), and mouse anti-CypA (1:1000, AbCam, Ab58144). Note that for more accurate quantification, the β-actin and CypA antibodies used in (B) were raised in different host species from those in (A) so that the residual signal left on the membrane from the first probing could be distinguished. For (A), the signal from the anti-rabbit secondary was used to quantify the CypA bands and that from the anti-mouse secondary for β-actin. For (B), the signal from the anti-mouse secondary was used to quantify the CypA bands and that from the anti-rabbit secondary for β-actin. The quantifications for these bands are shown in the respective Figure Supplements for the experiments where each construct was used. The source of the protein lysate run in each lane and size of the expected bands is listed in (C) in accordance with the numbers listed at the top of each membrane.

Supplementary file 2. Protein sequence similarity and identity matrices of PI4KA from select species.

Transparent reporting form
