# Supplementary figures and images for: Differences across cyclophilin A orthologs contribute to the host range restriction of hepatitis C virus
Source: eLife. 2019 May 10;8:e44436. doi: 10.7554/eLife.44436 (PMC6510530; doi:10.7554/eLife.44436)

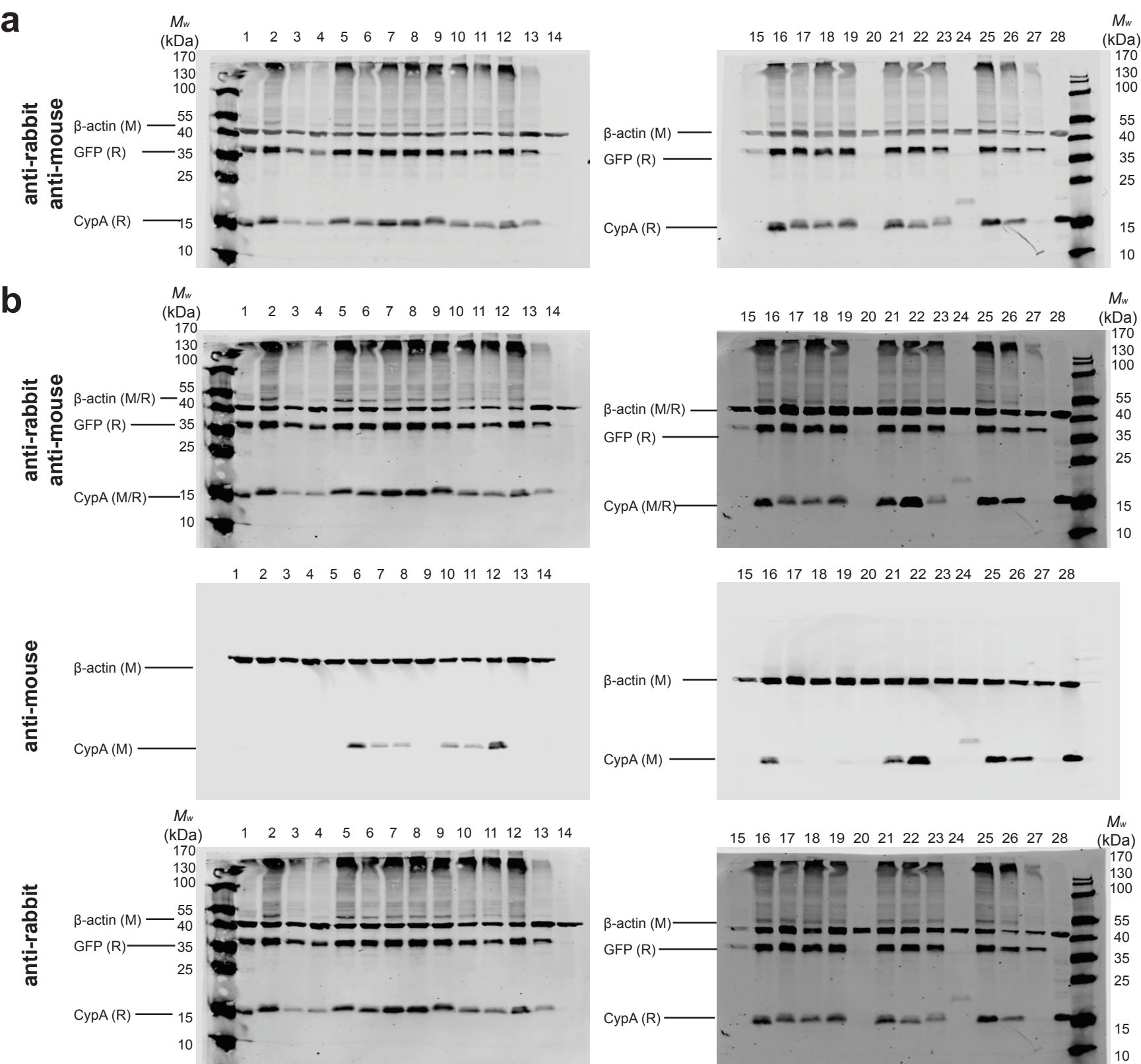

Supplement: Supplementary file 1. — All the constructs utilized in this paper were analyzed by western blot across two membranes. Shown here are the membranes after exposure to different combinations of antibodies, with the column on the left all images of one membrane and the column on the right images of the second. Whether the bands observed in a given set of membranes originated from the goat, mouse or both secondary antibodies is delineated at the left of each row. The proteins represented by the bands are also labeled, with ‘(M)’ and ‘(R)’ indicating signal from anti-mouse or anti-rabbit secondary, respectively. The blots shown in (A) were incubated with the following primary antibodies: rabbit anti-GFP (1:1000, Cell Signaling Technologies #2956S), mouse anti-β-actin (1:1000, Cell Signaling Technologies, #3700), and rabbit anti-CypA (1:1000, Cell Signaling Technologies #2175S). These blots were stripped and re-probed in (B) with the following primary antibodies: rabbit anti-GFP (1:1000, Cell Signaling Technolgoies #2956S), rabbit anti-β-actin (1:1000, 4970S), and mouse anti-CypA (1:1000, AbCam, Ab58144). Note that for more accurate quantification, the β-actin and CypA antibodies used in (B) were raised in different host species from those in (A) so that the residual signal left on the membrane from the first probing could be distinguished. For (A), the signal from the anti-rabbit secondary was used to quantify the CypA bands and that from the anti-mouse secondary for β-actin. For (B), the signal from the anti-mouse secondary was used to quantify the CypA bands and that from the anti-rabbit secondary for β-actin. The quantifications for these bands are shown in the respective Figure Supplements for the experiments where each construct was used. The source of the protein lysate run in each lane and size of the expected bands is listed in (C) in accordance with the numbers listed at the top of each membrane. [file elife-44436-supp1.pdf]
