# Supplementary material for: Differences across cyclophilin A orthologs contribute to the host range restriction of hepatitis C virus
Source: eLife. 2019 May 10;8:e44436. doi: 10.7554/eLife.44436 (PMC6510530; doi:10.7554/eLife.44436)
Supplement: Peer review history

# Decision letter

**DOI:** 10.7554/eLife.44436.021

**Affiliations:** Ulm University Medical CenterGermany; Ulm University Medical CenterGermany; Stanford UniversityUnited States; Scripps Research InstituteUnited States

In the interests of transparency, eLife includes the editorial decision letter and accompanying author responses. A lightly edited version of the letter sent to the authors after peer review is shown, indicating the most substantive concerns; minor comments are not usually included.

Thank you for submitting your article "Differences across cyclophilin A orthologs contribute to the host range restriction of hepatitis C virus" for consideration by *eLife*. Your article has been reviewed by three peer reviewers including Frank Kirchhoff as the Reviewing Editor and Reviewer #1, and the evaluation has been overseen by Dr. Anna Akhmanova as the Senior Editor. The following individuals involved in review of your submission have agreed to reveal their identity: Shirit Einav (Reviewer #2) and Philippe Gallay (Reviewer #3).

The reviewers have discussed the reviews with one another and the Reviewing Editor has drafted this decision to help you prepare a revised submission.

Summary:

In this study, Gaska and colleagues analyzed the role of the virus-dependency factor cyclophilin A (CypA) in the narrow host range of Hepatitis C virus (HCV). They show that CypA orthologs from most species investigated including mice only partially (30%) rescued Jc1-Gluc HCV replication in human CypA-knockdown Huh7.5-shRNA CypA cells compared to human CypA (100%). They further show that humanizing" murine CypA, by substituting three of six residues that differ between human and murine CypA orthologues rescue HCV replication at levels comparable to that of human CypA. They also found that the co-presence of human and murine CypA does not influence HCV replication, suggesting that murine CypA does not exhibit a dominant negative impact on viral replication. Finally, they generated a mouse-derived cell line expressing several human factors involved in the HCV life cycle and further transduced it with either human CypA, murine CypA or triply "humanized" murine CypA. After Jc1-Gluc HCV exposure, the authors found that the "humanized" murine CypA partially restored virion production of the "humanized" murine hepatoma cell line. However, viral production was still much lower compared to human Huh7 cells suggesting that additional species-specific restriction or viral dependency factors remain to be discovered.

The study addresses an important issue since an immunocompetent small animal model for HCV would be highly valuable for basic and clinical research. For most part, the manuscript is well written, experiments well controlled und the data are clearly presented and convincing. Limitations are that the rescue effect of humanized CypA on HCV replication is modest. In addition, the mechanism(s) underlying the species-specific differences of CypA to promote HCV replication remain unknown. Nonetheless, the findings are of significant interest. As outlined below some experiments require additional controls to warrant definitive conclusions.

Essential revisions:

1) Figure 1: Expression levels of the different CypA orthologues and mutants should be determined. Otherwise the specificity of functional differences is difficult to assess.

2) Figure 2B. It will be helpful to indicate not only the residues of the CypA ligand binding site but also those of the catalytic site (from my reading: H54, R55, F60, Q111, F113, and H126). Figures 2C-E: The authors tested the effect of overexpression of a triply murinized/humanized CypA on HCV RNA replication. Nevertheless, the three mutated residues were adjacent to each other, suggesting that they all may facilitate a similar role and thus their combination is less likely to result in a synergistic effect. To increase the likelihood of synergy, it will be important to combine the G52S mutation with 1 or 3 of the triple mutant and repeat these experiments. This may also help to see a phenotype in the murine cell line. The authors used an unpaired t test for statistical analysis in these panels. However, since multiple groups are being compared, ANOVA followed by a multiple comparison test should be used.

3) Figure 5: If the combination of the G52S mutant with one or three of the triple mutations is found to increase rescue in Huh7.5 cells (see Figure 2 above), it will be important to also test its rescue effect in Clone 8. As in Figure 2, ANOVA followed by a multiple comparison test should be used here.

4) Secreted Gaussia luciferase was used as indicator for HCV replication. This readout has the advantage that it is convenient and highly sensitive. However, the results are difficult to assess. Do the authors indeed measure replication (i.e. spreading infection) or just Gaussia expressed by initial infection? This should be discussed and, if feasible, key results should be verified by measuring infectious HCV yield. Infectivity data of the released particles from the "humanized" murine hepatoma cell line will also clarify whether or not there is a direct correlation between levels of virus production and levels of infectivity of de novo particles.

5) The function(s) of CypA in the HCV life cycle should be better presented. For example, to better describe the putative role(s) of CypA in the early steps of HCV replication (i.e., double membrane vesicle formation for efficient viral genome production and safety) as well as in the late steps of HCV replication, although a CypA role in the late stages of viral production remains controversial.

[Editors' note: further revisions were requested prior to acceptance, as described below.]

Thank you for resubmitting your work entitled "Differences across cyclophilin A orthologs contribute to the host range restriction of hepatitis C virus" for further consideration at *eLife*. Your revised article has been favorably evaluated by Anna Akhmanova as the Senior Editor, and a Reviewing Editor.

The manuscript has been improved but there are some remaining issues that need to be addressed before acceptance, as outlined below:

Our first point of the essential revisions was "Figure 1: Expression levels of the different CypA orthologues and mutants should be determined. Otherwise the specificity of functional differences is difficult to assess." In brief, you reply that you used "bicistronic constructs with CypA variants followed by an IRES-regulated GFP-ubiquitin-neomycin resistance fusion (in the case of the competition experiments where cells were dually transduced with both human and mouse CypA, the human CypA had a C-terminal 3x FLAG tag). We routinely assessed the CypA transduction efficiencies by flow cytometry prior to HCV infection, determining the percentage of eGFP+ (or FLAG+) cells […]" While your approach allows to assess the transduction efficiencies it does not provide definitive information on the expression levels of the different CypA orthologues and mutants and some might be unstable. To clarify whether the observed functional differences are specific you should determine the protein expression levels. It this is not feasible explain why and critically discuss this limitation. Notably, the first option is much preferred.

# Reply: Author response

**DOI:** 10.7554/eLife.44436.022

Essential revisions:

1) Figure 1: Expression levels of the different CypA orthologues and mutants should be determined. Otherwise the specificity of functional differences is difficult to assess.

For all experiments, we utilized bicistronic constructs with CypA variants followed by an IRES-regulated GFP-ubiquitin-neomycin resistance fusion (in the case of the competition experiments where cells were dually transduced with both human and mouse CypA, the human CypA had a C-terminal 3x FLAG tag). We routinely assessed the CypA transduction efficiencies by flow cytometry prior to HCV infection, determining the percentage of eGFP+ (or FLAG+) cells. We included representative data in Figure 1—figure supplement 1 but, we failed to cite this in the manuscript! We thank the reviewers for catching this oversight and have now corrected this error. For greater transparency of our experiments, we also expanded Figure 1—figure supplement 2; Figure 2—figure supplement 1; Figure 3—figure supplement 1; Figure 4—figure supplement 1) to include additional representative transduction efficiencies from the experiments performed throughout the paper.

2) Figure 2B. It will be helpful to indicate not only the residues of the CypA ligand binding site but also those of the catalytic site (from my reading: H54, R55, F60, Q111, F113, and H126).

We thank the reviewers for this recommendation. We have altered Figure 2B and the accompanying figure legend to be more specific, listing the residues which are known to directly interact with CsA and showing them in gray as sticks. With the exception of residue 54, all the active site residues also fall in this CsA-binding site. We specify these residues in black and also list them in the figure legend for clarity.

Figures 2C-E: The authors tested the effect of overexpression of a triply murinized/humanized CypA on HCV RNA replication. Nevertheless, the three mutated residues were adjacent to each other, suggesting that they all may facilitate a similar role and thus their combination is less likely to result in a synergistic effect. To increase the likelihood of synergy, it will be important to combine the G52S mutation with 1 or 3 of the triple mutant and repeat these experiments. This may also help to see a phenotype in the murine cell line.

We appreciate this recommendation from the reviewers and performed additional experiments now depicted in a new Figure 3 that is described in the Results section (subsection “Identifying the amino acid basis for the decreased ability of mouse CypA to facilitate HCV replication”, last paragraph). We combined the humanized mouse CypA mutant S52C that resulted in a significant gain-of-function of mouse CypA with each of the individual mutants T11A, A12V, and D14G as well as with the triply humanized mutant T11A/A12V/D14G (schematic in new Figure 3A). We assessed the replication efficiency for these mutants +/- S52C and did not observe a significant increase in rescue efficiency upon combining with S52C. Although there was an increase in rescue efficiency for the T11A/S52C mutant compared to T11A, this was not significantly higher than “wild-type” mouse and also did not exceed the triple T11A/A12V/D14G mutant.

The authors used an unpaired t test for statistical analysis in these panels. However, since multiple groups are being compared, ANOVA followed by a multiple comparison test should be used.

We thank the reviewers for their guidance and have re-performed all the statistics throughout our paper accordingly and added the detailed information regarding ANOVA and multiple comparison tests used in the respective figure legends.

3) Figure 5: If the combination of the G52S mutant with one or three of the triple mutations is found to increase rescue in Huh7.5 cells (see Figure 2 above), it will be important to also test its rescue effect in Clone 8. As in Figure 2, ANOVA followed by a multiple comparison test should be used here.

Based off the results of the recommended experiment shown in our new Figure 3, we did not move forward with the additional mutations in the Clone 8 line. We thank the reviewers once more for their keen eye regarding the statistical tests and as mentioned above, we have made the appropriate changes throughout our paper.

4) Secreted Gaussia luciferase was used as indicator for HCV replication. This readout has the advantage that it is convenient and highly sensitive. However, the results are difficult to assess. Do the authors indeed measure replication (i.e. spreading infection) or just Gaussia expressed by initial infection? This should be discussed and, if feasible, key results should be verified by measuring infectious HCV yield.

We are grateful for this insightful comment. We performed additional experiments to answer this question, resulting in a new Figure 5 (Results subsection “Triply humanized murine CypA supports HCV spread and release of infectious particles as efficiently as human CypA”). We assessed spread by both NS5A staining over time in select CypA rescue lines (new Figure 5B) and also by using supernatants collected from these infected cells at two different time points to subsequently infect naïve Huh7.5 cells to determine infectious particle release by analyzing Gluc activity in the supernatants of these cells (Figure 5C).

*Infectivity data of the released particles from the "humanized" murine hepatoma cell line will also clarify whether or not there is a direct correlation between levels of virus production and levels of infectivity of* de novo *particles.*

Similar to the experiments depicted in our Figure 4, we followed the reviewers’ recommendation to also look at infectious particle production from our Clone 8 lines. We took supernatants from infected Clone 8 cultures and used them to once more infect naïve Huh7.5 cells, which we then collected supernatants from for analyzing Gluc activity. As shown in the new panel of our previous Figure 4, now Figure 7C, particle production was very low with viral replication at 6 dpi (as represented by Gluc activity) not even a log above background.

5) The function(s) of CypA in the HCV life cycle should be better presented. For example, to better describe the putative role(s) of CypA in the early steps of HCV replication (i.e., double membrane vesicle formation for efficient viral genome production and safety) as well as in the late steps of HCV replication, although a CypA role in the late stages of viral production remains controversial.

In our efforts to keep the paper concise, we clearly were toobrief! We appreciate the reviewers’ identification of this weakness and have substantially expanded the Discussion to give a far more detailed overview concerning CypA and the HCV life cycle.

[Editors' note: further revisions were requested prior to acceptance, as described below.]

Thank you for resubmitting your work entitled "Differences across cyclophilin A orthologs contribute to the host range restriction of hepatitis C virus" for further consideration at eLife. Your revised article has been favorably evaluated by Anna Akhmanova as the Senior Editor, and a Reviewing Editor.

The manuscript has been improved but there are some remaining issues that need to be addressed before acceptance, as outlined below:

Our first point of the essential revisions was "Figure 1: Expression levels of the different CypA orthologues and mutants should be determined. Otherwise the specificity of functional differences is difficult to assess." In brief, you reply that you used "bicistronic constructs with CypA variants followed by an IRES-regulated GFP-ubiquitin-neomycin resistance fusion (in the case of the competition experiments where cells were dually transduced with both human and mouse CypA, the human CypA had a C-terminal 3x FLAG tag). We routinely assessed the CypA transduction efficiencies by flow cytometry prior to HCV infection, determining the percentage of eGFP+ (or FLAG+) cells…" While your approach allows to assess the transduction efficiencies it does not provide definitive information on the expression levels of the different CypA orthologues and mutants and some might be unstable. To clarify whether the observed functional differences are specific you should determine the protein expression levels. It this is not feasible explain why and critically discuss this limitation. Notably, the first option is much preferred.

We are grateful for the added clarification and the opportunity to add this to our manuscript. For all the CypA variants studied in our paper, we performed western blots to assess protein expression. Knowing that we were dealing with CypA variants of diverse sequence, we used two different primary antibodies with differing confirmed species reactivates. The only confirmed species covered by the two antibodies were human, mouse, rat, and African green monkey (which is 100% identical to human CypA at the amino acid level), and we were not able to find other antibodies listed as detecting squirrel monkey, pigtailed macaque or tree shrew. The western blots for all the CypA constructs contained in our paper can now be found in Supplementary file 1. We quantified the bands and show these data graphically in the appropriate figure supplements for the different sets of constructs (i.e. orthologs, murinized human CypA and humanized murine CypA single mutants, etc.).

It was clear that the reactivity of the antibodies we tested varied widely in their ability to detect the same construct, highlighting the difficulty to make precise comparisons in expression levels between transduced lines (i.e. 40-fold higher expression of construct A compared to the non-transduced Huh7.5 shRNA CypA is not necessarily equal to what is detected as 40-fold higher expression of construct B). We are comparing different constructs in the same line versus the same construct in different cell lines, so even if we had antibodies to specifically target each construct, these comparisons would remain problematic. This was one reason why we perceived a benefit in using a bicistronic system, so we could determine on a single cell basis how many cells theoretically contained our construct of interest.

In the end, of the 25 constructs tested, we were only unable to detect the pigtailed macaque TRIM5-CypA fusion and only weakly detected squirrel monkey CypA. We have discussed the ambiguity of our results for these two orthologs in both the Results and Discussion sections so that readers are clearly informed about the caveats. We also included broader discussion, much as written above, concerning the antibody reactivity issue that is unavoidable when working with such an array of constructs.
